# Response of the human gut and saliva microbiome to urbanization in Cameroon

**DOI:** 10.1038/s41598-020-59849-9

**Published:** 2020-02-18

**Authors:** Ana Lokmer, Sophie Aflalo, Norbert Amougou, Sophie Lafosse, Alain Froment, Francis Ekwin Tabe, Mathilde Poyet, Mathieu Groussin, Rihlat Said-Mohamed, Laure Ségurel

**Affiliations:** 1 UMR7206 Eco-anthropologie, CNRS - MNHN - Université de Paris, Paris, France; 2 Faculté de Médecine et des Sciences Biomédicales - Université Yaoundé 1, Yaoundé, Cameroun; 3 Department of Biological Engineering/Center for Microbiome Informatics and Therapeutics, Massachusetts Institute of Technology, Cambridge, MA USA; 4 The Broad Institute of MIT and Harvard, Cambridge, MA USA; 5 SAMRC/WITS Developmental Pathways for Health Research Unit, Department of Paediatrics, School of Clinical Medicine, Faculty of Health Sciences, University of Witwatersrand, Johannesburg, South Africa

**Keywords:** Microbial ecology, Microbiome

## Abstract

Urban populations from highly industrialized countries are characterized by a lower gut bacterial diversity as well as by changes in composition compared to rural populations from less industrialized countries. To unveil the mechanisms and factors leading to this diversity loss, it is necessary to identify the factors associated with urbanization-induced shifts at a smaller geographical scale, especially in less industrialized countries. To do so, we investigated potential associations between a variety of dietary, medical, parasitological and socio-cultural factors and the gut and saliva microbiomes of 147 individuals from three populations along an urbanization gradient in Cameroon. We found that the presence of *Entamoeba* sp., a commensal gut protozoan, followed by stool consistency, were major determinants of the gut microbiome diversity and composition. Interestingly, urban individuals have retained most of their gut eukaryotic and bacterial diversity despite significant changes in diet compared to the rural areas, suggesting that the loss of bacterial microbiome diversity observed in industrialized areas is likely associated with medication. Finally, we observed a weak positive correlation between the gut and the saliva microbiome diversity and composition, even though the saliva microbiome is mainly shaped by habitat-related factors.

## Introduction

Humans living in urban areas from highly industrialized countries host less diverse gut microbiomes than those in rural areas from the less industrialized ones. This effect of industrialization (as we refer to it hereafter) has been reported by numerous studies comparing urban areas in Europe or North America with rural ones in Africa, Asia or South America^1–8^. Loss in alpha diversity is further accompanied by systematic shifts in bacterial composition (see e.g.^9,10^). Despite crucial implications for human health^11,12^, the factors responsible for these ecological changes are still unclear. Two main (non-exclusive) hypotheses have been proposed: (i) decreased consumption of dietary fibers^13,14^ and (ii) improved sanitation and access to medical care (including antibiotics^15^). In addition, geographical latitude could be a confounding factor, given that it is a proxy for the diversity of environmental bacteria^16^. Indeed, the only two studies that did not find a loss of diversity with industrialization were studies considering rural populations not living in a warm tropical environment^17,18^.

To disentangle various factors potentially leading to this observed diversity loss, some authors focused on smaller geographical scales, contrasting urban and rural individuals from the same countries (referred to hereafter as the effect of urbanization). Such studies reported inconsistent results: no significant differences in Russia^19^ and Nigeria^20^, some differences in China^21^, and season and altitude dependent shifts in Mongolia^22^ and India^23^, respectively. These discrepancies can be partially explained by different definitions of rural areas used in these studies (from isolated pasturing areas in Mongolia to cities of 100,000 inhabitants in Russia) and by quite small sample sizes in some cases. Interestingly, whereas Winglee *et al*.^21^ observed, as expected, a trend towards higher OTU (operational taxonomic unit) and genus richness in rural areas, they found a higher evenness at higher taxonomic levels in urban areas (phylum, class and order). Therefore, urbanization seems to influence the gut microbiome diversity in a less clear-cut manner than industrialization, which is associated with a decrease in diversity regardless of the taxonomic resolution or the diversity measure (richness, evenness or phylogenetic diversity). This finding emphasizes the need to explore complex ecosystems such as human-associated microbiota using multiple diversity measures and taxonomic resolution levels, in order to capture complementary aspects of the studied microbial communities. More recently, Stagaman *et al*.^24^ investigated associations between economic factors and the gut microbiome diversity and composition in rural populations in Ecuador and showed that house modernity and power usage negatively correlated with the microbiome diversity. However, as the authors say, their economic metric could be a proxy for other lifestyle factors such as diet or healthcare practices. Estimating the relative importance of these factors thus requires a more comprehensive biological and cultural characterization of the populations under study.

In this regard, the work of Falony *et al*.^25^ represents a pioneer study, aiming to identify the factors associated with the variation in the gut microbiome composition in two large cohorts (1106 Flemish and 1135 Dutch subjects) among a large number (503) of covariates. They found that stool consistency, measured on the Bristol stool scale, had the strongest effect on the gut microbiome composition, while medication was the category explaining the largest amount of total variation. One question that emerges from this work is to which extent these results are transferable to other cultural contexts, for instance, to less industrialized populations, where the relationship between hosts and microbes has not been as much perturbed? Notably, highly industrialized populations have mostly lost gut protozoa^26^, whereas they are likely an important component of the gut ecosystem in less industrialized environments^27^. We have indeed previously shown that the presence of non-pathogenic gut protozoa from the genus *Entamoeba* is strongly correlated with an increase in gut microbiome diversity and shifts in composition^28^, and similar associations have been observed for *Blastocystis* in various countries^29–32^. However, it is not clear how the role of gut protozoa compares in magnitude with the factors identified in the work of Falony *et al*.^25^, as they are rare and thus cannot be properly studied in highly industrialized environments.

Finally, very little attention has been dedicated to the impact of urbanization and industrialization on other human microbiomes (e.g., oral, vaginal, skin). Answering this question could however help disentangling the effects that are due to diet (notably to fiber consumption), which should be specific to the gut, to general effects due to over-sanitized lifestyle, which could be shared across body sites. The few studies that have explored the influence of lifestyle on other microbiomes focused on the saliva microbiome and obtained conflicting results. Lassalle *et al*.^33^ observed a higher saliva microbiome diversity in rural Philippines than in the urban US, and Nasidze *et al*.^34^ found a higher diversity in rural hunter-gatherers from Uganda than in urban individuals from Sierra Leone and DRC, both of which are consistent with the results from the gut. However, African populations were characterized by a less diverse saliva microbiome than rural individuals from Alaska or urban individuals from Germany^35^, thus opposite to the trends observed for the gut microbiome. Similarly, the saliva microbiome of Native Americans in Oklahoma had lower diversity than the microbiome of neighbouring more urbanized individuals of European descent^36^. Finally, Clemente *et al*.^3^ found no difference between the saliva microbiome of the isolated Yanomami population from Venezuela and individuals from the US (despite differences in their gut microbiome diversity), suggesting no effect of lifestyle. Importantly and contrary to the gut, a more diverse saliva microbiome has been linked with a poorer oral health^37,38^.

**Figure 1:**
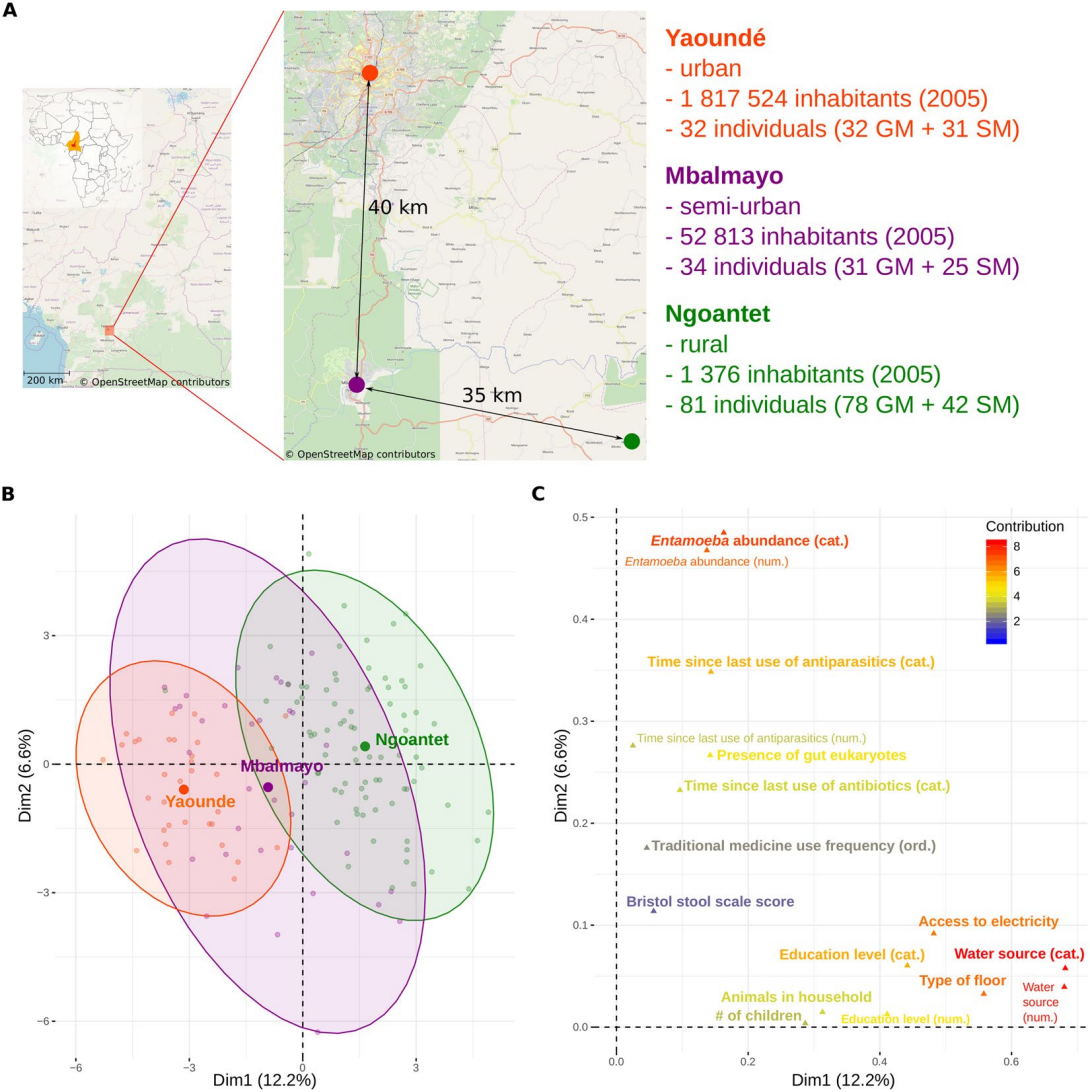
Description of the urbanization gradient. (**A**) Map of the sampling area^96^ and sampling design; numbers in brackets show the number of gut (GM) and saliva (SM) microbiomes included in the analysis. FAMD (Factor analysis of mixed data) of non-dietary contextual information showing (**B**) individuals colored by urbanization level (rural, semi-urban, urban) and (**C**) variables that significantly contributed to the construction of depicted axes; ordered factors were coded both as numerical (num.) and categorical (cat.) variable. Corresponding plots showing the 1^st^ and the 3^rd^ axis and the ordinations based on dietary can be found in Supplementary Figs. 1–3.

To examine the effect of urbanization on the gut and saliva microbiomes and to identify the factors associated with their variation in a less industrialized setting, we collected fecal and saliva samples of 147 individuals along an urbanization gradient in Cameroon (rural, semi-urban and urban populations, Fig. 1A). We further collected dietary, medical, parasitological and other socio-cultural information for each individual (Supplementary Table 1). We first described the urbanization gradient by identifying the factors that significantly varied across populations, and then we investigated changes in the gut and saliva microbiome diversity and composition based on the 16S rRNA gene amplicon sequencing data. We examined different aspects of diversity by calculating several indices and assessed the effect of OTU clustering level in order to explore multiple, potentially ecologically informative aspects of the data. This study is one of the first to collect a large number of variables describing biological and cultural variation (including diet, medical practices and other socio-economic factors) and to test them for association with diversity and composition of the human microbiome in a country with a minimum level of industrialization.

## Results

### The urbanization gradient is characterized by differences in diet, habitat and socio-cultural conditions, but not in medical practices or gut protozoa prevalence

In order to get a comprehensive overview of the biological and cultural factors associated with the sampled urbanization gradient (represented by a rural, a semi-urban and an urban population), we performed a multivariate ordination analysis for non-dietary information, dietary 24h-recall (quantifying the nutrients and energy intake in the last 24 hours) and dietary FFQ data (Food Frequency Questionnaire, assessing the average consumption of selected food categories over time) separately.

Non-dietary data included anthropometric traits, information on stool consistency and a number of parasitological, medical, sanitary and ethnographic variables (Supplementary Table 1). Ordination based on these non-dietary variables (Fig. 1B,C, Supplementary Fig. 1) revealed the urbanization gradient along the first ordination axis (R2 = 0.67, p < 10^−6^), which explained 12.2% of the variability and was associated with habitat-related and other socio-cultural factors (education level, number of children). Specifically, increasing urbanization was characterized by access to electricity, use of tap water as the primary water source and by the presence of cement floor and absence of animals in the house. Urban individuals also attained higher education level and had less children. The second and the third axis explained 6.6% and 6.5% variability respectively, and did not separate samples according to the urbanization level. On the other hand, the second axis distinguished the individuals based on the load of *Entamoeba* spp. (the most prevalent gut protozoan found in the parasitological analysis, with an overall prevalence of 45%) and the Bristol scale score (describing the stool consistency). Both the second and third axis could further be linked to a variety of medical factors (use of antibiotics, antiparasite drugs and traditional medicine) and smoking.

For the dietary 24h-recall data, the first three PCA axes did not well represent the urbanization gradient, although the third axis was weakly correlated withit (Supplementary Fig. 2, Axis 3 Anova: R2 = 0.13, p = 0.001). On the other hand, PCA on the FFQ data (not collected for the semi-urban population) resulted in a clear separation of rural and urban samples along the first axis explaining 10.7% of the total variability (Supplementary Fig. 3, Anova R2 = 0.48, p < 10^−6^). The variables associated with this axis were overall concordant with those identified by the *v* test as differentially represented in the rural and urban population (Supplementary Fig. 3E). Specifically, urban individuals eat more dairy products (industrial yoghurt, kossam, milk), more animal-based food (meat, chicken, insects), as well as more sweets and soft drinks. Rural individuals, on the other hand, eat more root crops (cassava, cocoyam), leaf vegetable and peanuts, but also drink more palm wine.

Overall, the studied urbanization gradient can be mainly described in terms of habitat-related factors (such as sanitary conditions), education level and number of children, as well as by differences in diet, particularly in the amount of processed food, meat and fibers. On the other hand, the prevalence of gut eukaryotes and medical practices seem to be mostly independent of the urbanization level.

### Gut microbiome diversity is shaped mainly by gut protozoa and stool consistency

**Figure 2:**
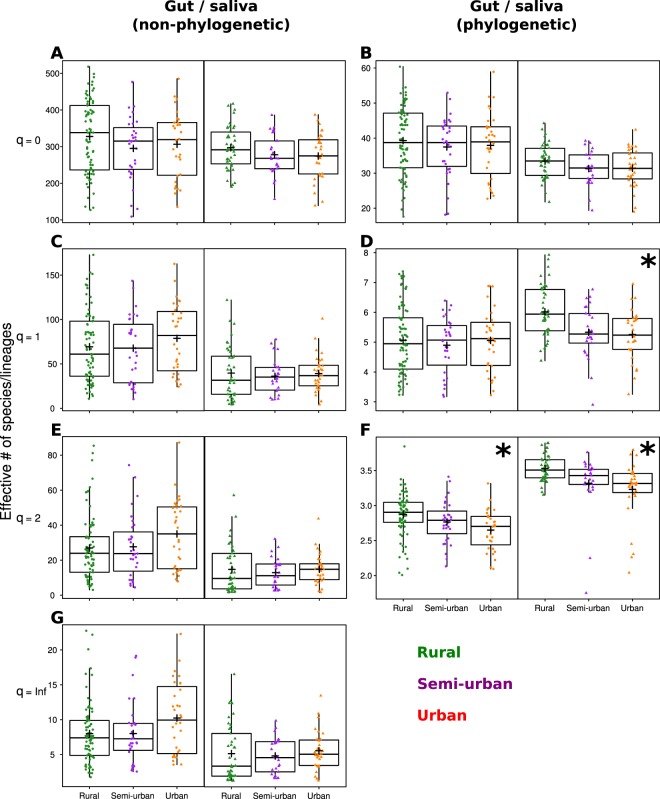
Alpha diversity of the gut and saliva microbiome along the urbanization gradient. Effective number of ASVs (**A**,**C**,**E**,**G**; non phylogenetic indices) and lineages (**B**,**D**,**F**; phylogenetic indices). Asterisk denotes significant directional changes along the gradient. ^+^Indicates the group mean.

To examine different aspects of non-phylogenetic and phylogenetic alpha diversity, we calculated weighted and non-weighted effective numbers of species or lineages (Table 1). None of the non-phylogenetic diversity measures (^0^D, ^1^D, ^2^D or ^Inf^D) nor the phylogenetic lineage richness (^0^D_ph_) changed along the urbanization gradient (Fig. 2, Supplementary Table 2). Conversely, dominant lineage diversity (^2^D_ph_) decreased by 6% with urbanization level in both the gut and the saliva microbiome. For the saliva microbiome, we also found a 10% decrease in common lineage diversity (^1^D_ph_) with increasing urbanization.

**Table 1:** Alpha diversity indices used in this study.

Index notation	Order	Phylogenetic	Interpretation
^0^D	0	no	not weighted by abundance = species richness
^1^D	1	no	species weighted exactly by their abundance = exp(Shannon’s H)
^2^D	2	no	more weight given to dominant species = inv. Simpson index
^Inf^D	Inf	no	only the most dominant species counts = inverse of Berger-Parker index
^0^D_ph_	0	yes	lineage richness = Faith’s PD
^1^D_ph_	1	yes	lineages weighted by their abundance (“richness of common lineages”)
^2^D_ph_	2	yes	more weight given to dominant lineages (“richness of abundant lineages”)

Order (q) defines how much weight is given to abundant taxa. See Methods for more details.

**Figure 3:**
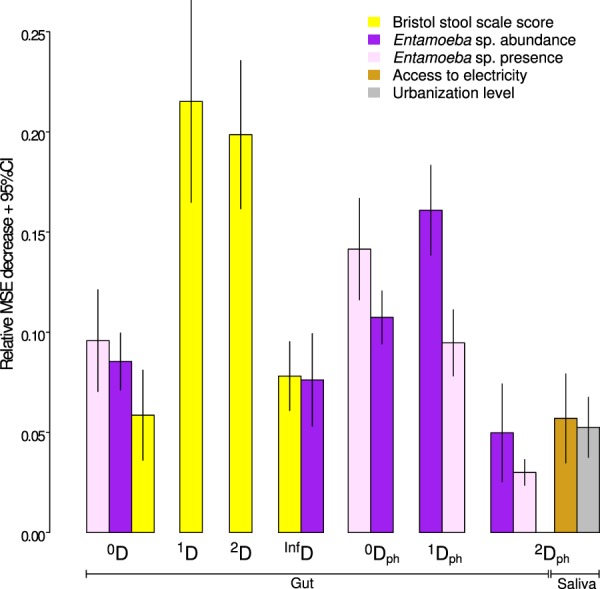
The most important predictors of ASV diversity for the gut and saliva microbiome selected by random forest regression. The values represent the means of 10 model replicates. Depicted variables were selected by kmeans clustering of the variable importance values as described in Methods section, only for the models with R-squared > 0.1. Both non-phylogenetic (D) and phylogenetic (D_ph_) diversity indices are shown. The order (^0^, ^1^, ^2^ and ^Inf^) represents the weight given to taxa abundances. Detailed description of diversity indices can be found in Table 1 and Methods. Corresponding plots showing the alpha diversity predictors for the rural-urban data subsets can be found in Supplementary Fig. 4. Complete results are in Supplementary Table 3.

We then identified the best predictors of alpha diversity by random forest regression. Depending on the diversity index, the models explained between 12% and 35% of variability in the gut microbiome diversity (Supplementary Table 3, R-squared test values). The most prominent predictors of the gut microbiome diversity were the abundance and/or presence of *Entamoeba* sp. (for all indexes except ^1^D and ^2^D) and the Bristol scale score (for all non-phylogenetic indexes, Fig. 3). Specifically, diversity increased in the presence of *Entamoeba* sp. and decreased with the Bristol scale score. Given that the load of *Entamoeba* sp. did not significantly decrease with urbanization, the observed urbanization-associated loss of the dominant lineage diversity (^2^D_ph_) in the gut microbiome is likely linked with another, unknown variable(s).

**Figure 4:**
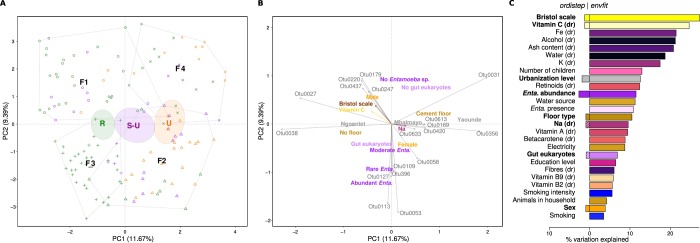
Gut microbiome composition. PCA showing: (**A**) individuals colored by urbanization level (rural, semi-urban, urban) and with shapes corresponding to individual enterotypes (F1–F4); (**B**) contextual variables selected by *ordistep* and ASVs associated either with these variables or with the ordination axes (only the ASVs and variables within the lower or upper 2.5% quantile are shown, for all variables see Supplementary Tables 7 and 8); arrows denote ASVs or numerical variables, factors are represented by their levels’ names placed at the centroid of individuals of the given category; (**C**) variation in community composition explained by different factors selected by *envfit* (right, each variable tested independently) and *ordistep* (left, the factors retained in the best non-redundant model); dr = 24 h-recall. Corresponding plots for the 1^st^ and 3^rd^ axis and for the rural-urban data subsets are in Supplementary Fig. 6.

As the dietary FFQ data were only available for the rural and urban samples, we performed additional analyses excluding the semi-urban samples, both with and without the dietary FFQ data. These analyses overall confirmed the above trends despite some minor differences (Supplementary Table 3, Fig. 4).

### Saliva microbiome diversity is shaped by habitat-related factors

For the saliva microbiome, only the models predicting ^2^D_ph_ (complete dataset) and ^1^D_ph_ (rural-urban subsets) had R-squared values >= 0.1 and were thus considered for interpretation (Fig. 3, Supplementary Table 3, Fig. 4). The strongest predictors of ^2^D_ph_ in all three data subsets were urbanization level and access to electricity. In the rural-urban datasets, ^1^D_ph_ and ^2^D_ph_ were additionally predicted by water source and the type of floor in the house (Supplementary Table 3, Supplementary Fig. 1). As both of these variables and access to electricity were strongly correlated with urbanization in pairwise comparisons (Supplementary Fig. 1, Supplementary Table 4), the observed decrease in saliva microbiome phylogenetic diversity (^1^D_ph_ and ^2^D_ph_) with urbanization (Fig. 2, Supplementary Table 2) might be linked to the differences in habitat-related factors.

### Gut microbiome composition is primarily shaped by gut protozoa, stool consistency and urbanization

**Figure 5:**
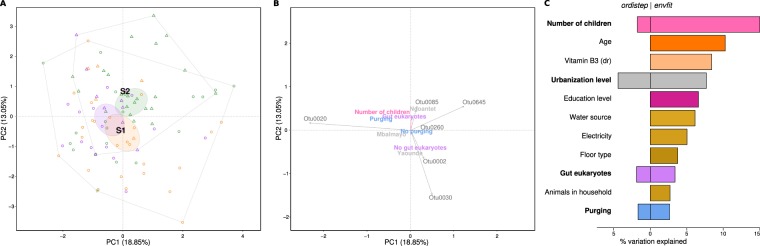
Saliva microbiome composition. PCA showing: (**A**) individuals colored by urbanization level (rural, semi-urban, urban) and with shapes corresponding to individual stomatotypes (S1 & S2); (**B**) contextual variables selected by *ordistep* and ASVs associated either with these variables or with the ordination axes (only the ASVs and variables within the lower or upper 2.5% quantile are shown, for all variables see Supplementary Tables 7 and 8); arrows denote ASVs or numerical variables, factors are represented by their levels’ names placed at the centroid of individuals of the given category; (**C**) variation in community composition explained by different factors selected by *envfit* (right, each variable tested independently) and *ordistep* (left, the factors retained in the best non-redundant model); dr = 24 h-recall. Corresponding plots for the 1^st^ and 3^rd^ axis and for the rural-urban data subsets are in Supplementary Fig. 6.

We aimed both to detect the taxa whose relative abundances changed along the urbanization gradient and to identify the biological and cultural factors that best explain the variability of the gut microbiome. To accomplish the first goal, we analyzed the community composition with ALDEx2 (a tool for the differential abundance analysis of compositional data) and found changes in the relative abundance of 41/282 tested ASVs (Amplicon Sequence Variants) along the urbanization gradient (Supplementary Table 5). Whereas the relative abundance of some genera either consistently decreased (e.g. *Succinivibrio*, *Faecalibacterium*, *Prevotella*, *Roseburia*, *Coprococcus*) or increased (e.g. *Allistipes*, *Bacteroides*, *Clostridium*, *Butyricicoccus*) with increasing urbanization, various unclassified *Lachnospiraceae* ASVs responded differently to urbanization. In order to identify phylogenetic lineages that are most consistently affected by urbanization regardless of the phylogenetic and taxonomic scale, we performed phylofactorization. The majority of phylofactors were composed of a single ASV, indicating that urbanization-related changes occurred primarily at a fine phylogenetic scale (Supplementary Table 6). Interestingly, 16 phylofactors were composed of one or more *Prevotella* ASVs that all had consistently higher abundance in rural samples, suggesting that different strains disappear at different rates in response to urbanization. Detailed phylofactorization results can be found in Supplementary Table 6, Fig. 5 and Supplementary results.

To identify the factors that best explain variation in the composition of the gut microbiome, we performed a PCA on the Euclidean distances of *clr-*transformed data (the equivalent of Aitchison distance, taking into account the compositionality of the sequencing data), followed by single variable testing (*envfit*) and forward-backward non-redundant variable selection (*ordistep*). The first three PCA axes explained together 24.9% of the variability in the gut microbiome composition (Fig. 4, Supplementary Fig. 6). Despite the large within-group variation, the urbanization gradient was apparent along the first axis together with the associated ASVs, largely consistent with ALDex2 results (Supplementary Table 5). The second axis distinguished the samples according to *Entamoeba* sp. abundance; the ASVs with the highest coordinates on the second axis (associated with the samples negative for *Entamoeba* sp.) belonged to *Suterella*, *Bacteroides*, *Alistipes* and *Prevotella*, whereas the ASVs with the lowest score on the second axis (associated with *Entamoeba* carriage) were assigned to *Butyrivibrio* and unclassified *Clostridiales*, *Ruminococcaceae*, *Lachnospiraceae* and *Firmicutes* (Supplementary Table 7). Although *envfit* identified 27 variables as significantly associated with the gut microbiome variation, with the Bristol scale score and several 24h-recall variables at the top, only eight were kept by the *ordistep* forward-backward selection in the final non-redundant model explaining 21% of total variability. These were, ordered by the effect size: *Entamoeba sp*. abundance, urbanization level, Bristol scale score, recent intake of vitamin C, sex, type of floor, and amount of recently ingested Na (Fig. 5C, Supplementary Table 8). The analysis of rural-urban data subsets yielded overall concordant results, with some differences, reported in Supplementary Fig. 6 and Tables 7 and 8. It is noteworthy that, if FFQ data were included in the analysis, *ordistep* retained - among other - the average consumption of palm wine and meat, but not the urbanization level. The fact that the urbanization level was retained in the analysis without the FFQ data suggests that urbanization level at least partially tags dietary differences between populations.

Finally, we identified four enterotypes (gut community types),: F1, strongly dominated by *Prevotella*; F2, enriched in *Ruminococcaceae*, unclassified *Clostridiales*, *Lachnospiraceae* and another unclassified bacterial genus; F3, which could be considered as a transient type between the first two; and F4, dominated by *Bacteroides* and *Lachnospiraceae* (Supplementary Fig. 7). These were differentially distributed between the studied populations, with 74% of rural individuals characterized by enterotypes F1 and F3, 74% of semi-urban individuals by enterotypes F1 and F2, and 59% of urban individuals by the enterotype F2 (Fisher’s exact test < 0.05). A more detailed description and the analysis of enterotypes can be found in the Supplementary results and Supplementary Fig. 8.

### Saliva microbiome composition is influenced by urbanization

Regarding the saliva microbiome, the relative abundance of only eight out of 110 ASVs tested by ALDex2 significantly changed along the urbanization gradient (Supplementary Table 5). Specifically, urbanization was accompanied by an increase in the relative abundance of three ASVs from the genera *Veillonella*, *Rothia*, *Streptococcus* and *Actinomyces* and a decrease in the relative abundance of two ASVs assigned to *Fusobacterium* and unclassifed *Pasteurellaceae*. Phylofactorization identified 26 edges as significantly differentiating between the three populations, with nine phylofactors composed of more than one ASV (Supplementary Table 6, observed and predicted changes in relative abundances associated with the first six phylofactors can be found in Supplementary Fig. 5).

The first three PCA axes explained 46% of the variability in the saliva microbiome composition (Fig. 5). The first axis corresponded to a gradient from two *Prevotella* and one *Veillonella* ASVs to an unclassified *Firmicutes* ASV and did not well represent either urbanization or other variables. On the other hand, the second and the third axis partially distinguished between rural and urban samples, with *Enterobacteriaceae* (2^nd^ axis) and *Prevotella* (3^rd^ axis)enriched in the rural population (Supplementary Table 6). One-way tests retained 11 variables, with the number of children and age explaining the highest amount of variability, whereas only four factors were kept in the selected non-redundant model: urbanization level, presence of gut eukaryotes, number of children and purging (Fig. 5C). The results based on rural-urban dataset were different: if FFQ variables were included, *ordistep* kept the average consumption of soya and cassava sticks, type of floor, presence of animals in the house and type of toilets; without the FFQ variables, only the sanitary conditions were retained (Supplementary Fig. 6).

Saliva microbial communities in the studied populations could be grouped into two types (stomatotypes): S1, dominated by *Prevotella* and *Streptococcus* and S2, enriched in *Enterobacteriaceae*. The urban population, with 84% of individuals hosting the S1 stomatotype, significantly differed from the semi-urban and rural population, where both stomatotypes were equally represented (Fisher’s exact test, rural - urban: p = 0.005, semi-urban - urban: p = 0.054, Supplementary Fig. 7). More details can be found in Supplementary results.

### Relationship between the gut and saliva microbiome

**Figure 6:**
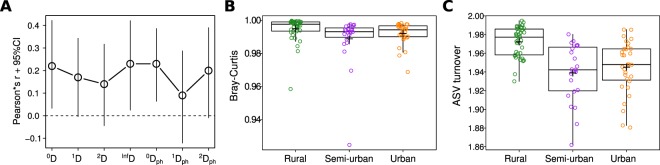
Relationship between gut and saliva microbiome. (**A**) Correlation between ASV alpha diversity measures (summarized in Table 1) between gut and saliva microbiomes of the same individual (n = 95); (**B**) average Bray-Curtis dissimilarities and (**C**) average ASV turnover (corrected for differences in species richness, see Methods) between gut and saliva microbiome of the same individual, for each population separately. Cross denotes mean.

To examine the relationship between the gut and saliva microbiome, we first calculated Pearson correlations between the alpha diversity values within each individual. We found weak positive correlations between the species and lineage richness (^0^D and ^0^D_ph_) as well as ^Inf^D of the gut and saliva microbiome (Pearson r = 0.23 and r = 0.22, respectively, Fig. 6, Supplementary Fig. 9). We then asked if the gut and saliva microbiome of an individual shared more species than a random gut-saliva microbiome pair. This was indeed the case (Supplementary Fig. 10, standardized effect = −0.30, p = 0.003,), but the effect became only marginally significant when permutations were restricted to within urbanization levels (standardized effect size = −0.14, p = 0.052). Subsequently, we checked if the similarity between the gut and saliva microbiome varied across the urbanization levels. We found that the gut and saliva microbiomes of rural individuals shared slightly less ASVs than the microbiomes of semi-urban and urban individuals (Fig. 6, F2, 92 = 19.95, p < 10^−8^, 0.98% decrease). Conversely, we found no significant influence of the urbanization level on Bray-Curtis dissimilarities between the saliva and gut microbiome, despite a decreasing trend (F_2,92_ = 2.96, p = 0.056). Additional analyses exploring the effect of OTU clustering on the conclusions of the study can be found in the Supplementary Figs. 11–13 and Supplementary results.

## Discussion

By using detailed biological and cultural characterization of the populations under study, we show that the presence of a commensal gut eukaryote *Entamoeba* sp. is the strongest determinant of gut microbiome diversity and composition in a minimally industrialized country. Gut protozoa thus appear as an important factor to consider when studying the ecology and evolution of the gut ecosystem. As the studied populations did not differ in either *Entamoeba* sp. prevalence, gut microbiome richness or medical practices (despite the differences in diet), we propose that the loss of microbiome diversity in highly industrialized countries might be partially explained either by (i) the loss of gut eukaryotes or (ii) differences in medication.

Whereas the direction of compositional shifts (such as decrease in *Succinivibrio* or *Prevotella*, and increase in *Bacteroides* or *Allistipes*) along the urbanization gradient was consistent with those previously observed when comparing populations with different industrialization^1,3,5^ or urbanization^20,21,23,39^ levels, we found little evidence of diversity loss, which usually accompanies industrialization^1–8^. Together with other studies reporting inconsistent findings regarding the difference in diversity between neighbouring rural and urban populations^20,21,23^, this suggests that the bacterial diversity loss might mostly occur between urban areas from minimally industrialized countries and urban areas from highly industrialized countries. Interestingly, this might also be the case for commensal gut eukaryotes, as the prevalence of *Entamoeba* sp. did not differ significantly between our urban and rural areas. Industrialization is thus accompanied both by a loss of bacterial gut microbiome diversity and by disappearance of *Entamoeba* spp. (both changing only slightly along the urbanization gradient studied here), suggesting that these two processes could be directly or indirectly related to each other.

Knowing that the presence of *Entamoeba* sp. is the strongest determinant of the gut microbiome diversity, it is indeed possible that the loss of gut eukaryotes might partially explain the loss of bacterial diversity in highly industrialized countries. Although most species of the genus *Entamoeba* are non-pathogenic and seem to be common residents of the healthy human gut in developing countries^26^, very little is known about their global prevalence^40^, and even less about their relationship with the gut bacterial microbiome^27,41^. In addition, none of the previous studies focusing on the effects of urbanization or industrialization examined diversity of the protozoan symbionts. Alternatively, as our urbanization gradient mostly represents changes in terms of diet and habitat, but not medication, is is tempting to propose that the loss of both bacterial and eukaryotic diversity in highly industrialized and urban environments is mostly due to changes in medical practices, such as antibiotics use. Finally, we did observe a decline in abundance-weighted phylogenetic diversity (^2^D_ph_), which is more sensitive to subtle changes in environmental conditions than richness measures^38^. This small but significant decrease might represent an early response to urbanization-related changes in lifestyle, for example due to changes in diet.

Our observation that the stool consistency is another strong predictor of gut microbiome diversity and composition is consistent with previous findings from industrialized populations^25,42^. Interestingly, although the stool consistency explained the largest portion of variability in the gut microbiome composition when tested separately, it was only the third best factor in the final redundant model, probably because of interactions and correlations between the tested covariates. Strong correlations between the dietary variables, which had the second largest combined effect on the gut microbiome composition, could also explain why the exact selected variables vary between the data subsets (complete vs. rural-urban). For example, the intake of vitamin C was strongly correlated with the amount of alcohol, which is more likely to have an effect based on the findings from industrialized populations^25^, but the former variable was selected as the most important. Finally, the inclusion of FFQ data, describing the average diet, resulted in the exclusion of the urbanization level from the non-redundant models for the rural-urban data subset, suggesting that the urbanization-related differences in composition could be to a large extent explained by dietary shifts, contrary to the findings of Stagaman *et al*.^24^.

Human saliva and gut are ecologically distinct habitats and we were surprised to observe an urbanization-related decrease in abundance-weighted phylogenetic diversity (describing community structure) for both gut and saliva microbiome, considering that each is shaped by different set of factors. Therefore, this decrease seems to be associated with different processes in each case rather than with a common response of the two microbiomes to the changes in lifestyle. Similarly, it is interesting that we observed a positive relationship between the species (and lineage) richness of the two microbiomes, considering that the diversity of the gut and saliva microbiome seems to be related to health in opposing ways^11,38^. Still, this positive correlation could be related to the degree to which individuals are exposed to environmental bacteria. The higher proportion of shared species within individual than in a random gut-saliva pair indeed provides some support for the role of the environment as a source of colonization for the human microbiome. Interestingly, the proportion of shared species within indvidual decreased with the urbanization level (Fig. 6), suggesting a difference in dispersal dynamics between the populations as well as different effect of urbanization on the factors shaping the rare portion of each of the two microbiomes. Although seeding from oral microbiome seems to occur normally in healthy humans^43,44^, excessive colonization of the gut by oral bacteria has been associated with disease^45^. It is noteworthy that we observed different proportions of shared species depending on the enterotype, suggesting that enterotypes might differ in their resistance to colonization by external bacteria.

In our analysis, we were faced with challenges due to complexity of the dataset, comprising many correlated and interacting variables. In our decisions regarding e.g. appropriate thresholds for the variable selection in the random forest regression or definition of strong correlations, we chose to be strict in order to minimize false positives and avoid over-interpretation. Potential issues with correlations and interactions are well illustrated by the factors associated with the saliva microbiome composition: whereas it is conceivable that habitat and sanitary conditions^46^, diet^33^ or age^47^ shape the saliva microbiome, the fact that purging, gut eukaryotes and number of children are among the top predictors is puzzling. It is thus likely that at least some of these factors tag other correlated (known or unknown) variables or variable interactions. Despite these issues, by quantifying the correlations between the variables and by using (where possible) a random forest regression method that can deal with correlated predictors^48,49^, we were able to identify potentially important determinants of gut and saliva microbiome diversity and composition in a minimally industrialized country. In order to improve our understanding of the factors that shape the human microbiome and to resolve the correlations between them, it is necessary to examine and understand the role of individual factors in controlled studies, including studies on animal models. This should be complemented by the studies with a sampling design such as in^33^ or^19^, with multiple urbanization/industrialization gradient replicates, and by collecting a wide variety of contextual data, as done here and in Stagaman *et al*.^24^.

In conclusion, urbanization was associated with minor shifts in diversity of the gut and saliva microbiome, but also with changes in the gut microbiome composition that were reminiscent of those associated with industrialization. Although some of the important factors shaping the gut microbiome, such as stool consistency and diet, are the same as in industrialized populations, gut eukaryotes (*Entamoeba* sp.) represent the major determinant of diversity and composition here. This may be one of the main confounders when comparing industrialized (virtually gut-eukaryote free) and non-industrialized populations. In order to advance our understanding of how industrialization affects the human microbiome, we need to account for these confounders, for example by routinely collecting more cultural data as well as by including parasitological information. Luckily, microscopic analyses of the fecal samples are not necessary if metagenomics data is collected, as parasitological screening can be performed from genomic data^26^.

## Methods

### Ethics approval and consent to participate

The research permits, including the appropriate ethic approvals, were obtained for this study from the CNERSH (Comité national d’Ethique de la Recherche pour la Santé Humaine) in Cameroon (Approval N°2017/05/900), as well as from regional health districts (Centre region, Approval N°0061). We further obtained an ethical approval from the French CPP (Comité de Protection des Personnes, approval N°2016-sept-14344), as well as the authorization to import and store these samples from the French Ministry of Higher Education and Research (N°IE-2016-876 and DC-2016-2740, respectively). Finally, we obtained the authorization to store personal data in France from the CNIL (Commission Nationale Informatique et Libertés, N°1972648). We obtained the informed consent of each participant for contributing to this research and publishing the associated-data. Consent for publication is not applicable. All research was performed in accordance with relevant guidelines and regulations.

### Sampling and collection of contextual data

We sampled 147 adult volunteers at three locations in Cameroon (Fig. 1A), representing an urbanization gradient: 81 individuals in Ngoantet (rural), 34 in Mbalmayo (semi-urban) and 32 in Yaoundé (urban), after obtaining their informed consent. The participants declared being currently healthy, i.e. not taking medication for any infectious or metabolic disease or suffering from specific symptoms (e.g. fever). They were between 18 and 65 years old and were not related at the second degree. They self-collected the feces during the morning in sterile recipients provided by us (Medline stool containers). We then placed app. 3 g of the fecal samples into 60 mL containers with RNAlater. For saliva, the participants spit 2 mL of saliva into a 15 mL falcon tube, to which we added the same volume of a homemade buffer (5 mM TRIS, 5 mM EDTA, 5 mM sucrose, 10 mM NaCl, 1% SDS, pH = 8).

In addition, we recorded contextual information (metadata) about the participants (Supplementary Table 1) in collaboration with local investigators and trained research assistants. Specifically, we collected dietary information using a quantitative 24-hour recall questionnaire (recording the amount of all the items the individual has eaten during the last 24 hours, which is then transformed into the amounts of nutrients and energy ingested) and a qualitative food frequency questionnaire (FFQ, reporting how often the selected types of food were consumed during the last month^50^). The FFQ data were collected only for the rural and urban population. Non-dietary information including medical (use of antibiotics, antiparasitics, and traditional medicine) and other socio-economic and socio-demographic variables (water source, type of floor, animals in household, access to electricity, smoking, number of children, education) were collected using questionnaires. Only the animals that were declared by the participants to live in the house were considered as the animals in the household and included: cats, dogs, guinea pigs, parrots, goats, pigs, ducks and poultry. We did not consider the presence of mice. For smoking habits, we grouped individuals into five groups: non-smokers (“0_non”), “1_recent”, who started smoking during the last year, “2_medium”, who have smoked for less than 5 years, “3_longtime” - smoking for 5+ years, less than 10 times a day and “4_longtimeHeavy” - smoking for 5+ years, 10 or more times a day.

Anthropometric traits (height and weight) were measured using standardized techniques. The Centre Pasteur du Cameroun furthermore performed a microscopic analysis of the fecal samples to identify the gut eukaryotes (with a bias toward the identification of potentially pathogenic species, i.e., worms and *Entamoeba* sp.). For *Entamoeba* sp., we grouped individuals in categories depending on the output of the microscopic analysis as follows: “0_ none” if no cyst nor any trophozoites was seen; “1_rare” if only rare cysts were observed; “2_some” if only few cysts or few trophozoites were observed; “3_abundant” if numerous cysts and/or trophozoites were observed. Finally, we estimated the Bristol stool scale score by visually assessing the stool consistency (a proxy for colonic transit time and water content^51^).

### DNA extraction and sequencing

App. 250 mg of fecal material was disrupted by bead-beating and DNA was subsequently extracted with the MOBIO PowerFecal DNA isolation kit (MOBIO Laboratories, Carlsbad, CA, USA) according to the manufacturer’s protocol. DNA from the saliva samples was extracted following the protocol of Quinque *et al*.^52^. The extracted DNA was quantified with NanoDrop (ThermoScientific). Sequencing libraries targeting the V4 region of the 16S rRNA gene were prepared by the MOC core facility at the Broad Institute, Cambridge, MA, USA and sequenced as 150 bp paired-end reads in two runs on an Illumina MiSeq. 300 (as described in^53^), resulting in an average of 61,617 ± 43,997 (st. dev.) raw reads per sample (Supplementary Table 9).

### Data analysis

All analyses were performed in R^54^ except for the parts of sequencing data preprocessing that was done in *mothur*^55^.

#### Metadata analysis

We imputed missing values in the contextual data using the *missForest* R package^56^. In order to assess the strength of pairwise correlations between the variables, we calculated Pearson’s r for pairs of numerical variables, Cramer’s V for categorical predictors (using the *rcompanion* package^57^ and omega for numerical-categorical combinations. We disregarded all correlation coefficients for which the t-test, chi-square test or Kruskal-Wallis test (for numerical, categorical and numerical-categorical variables, respectively) were not significant at alpha = 0.05. In addition to the urbanization level variable, we analyzed 51 variables collected in all three populations: 28 dietary variables from a 24h-recall questionnaire and 23 non-dietary variables. We analyzed 49 additional dietary FFQ variables for the urban and rural populations only. The presence of *Entamoeba* sp. and smoking habits were encoded both as binary, as well as ordered factors (specifying *Entamoeba* sp. load and smoking intensity and duration), which resulted in a total of 25 non-dietary variables tested for correlations. After correcting for multiple testing, 403 out of 1431 (28%) and 555 out of 5253 (11%) pairwise correlations between these variables were significant in the complete and rural-urban dataset, respectively (alpha = 0.05, Supplementary Table 4). Strong correlations (abs(Pearson) >= 0.8, Cramer’s V or Omega >= 0.4) were mainly found between 24h-recall dietary variables and between habitat-related (e.g. sanitary) variables (Supplementary Fig. 1). In addition, the urbanization level was associated with some habitat-related variables, including water source, access to electricity, type of floor and presence of animals in the house. The inclusion of the dietary FFQ data (and omission of semi-urban samples) revealed some additional correlations, notably between the urbanization level (or some of the associated habitat-related variables) and: education level, number of children and consumption of meat, cassava and peanuts.

In order to explore the multivariate aspects of the contextual data and to identify the factors that best describe the urbanization gradient, we used the *FactoMineR* package^58^. We first performed a factor analysis of mixed data (FAMD) with all non-dietary variables. The categorical variables representing the ordered factors were duplicated and one version was coded as a numerical while the other was coded as a categorical variable. We performed a separate PCA on 24h-recall dietary data normalized by total energy input, as well as on the FFQ data for the rural-urban data subset only. In each case, the correlation coefficient between the ordination axes and urbanization level were calculated with *dimdesc* in order to statistically test the representation of the urbanization gradient by the axes. In addition, we tested the association between FFQ data and urbanization level with the *v* test from the same package (function “catdes”).

#### Sequencing data quality control, taxonomy assignment and OTU clustering

Sequences were denoised using *dada2*^59^ according to the authors’ recommendations. First, the reads were processed with the *filterAndTrim* command in order to remove phiX spike-in and reads with ambiguous bases, to truncate reads at the first base with quality score <= 2 and to filter reads with more than 2 expected errors. The sequences were then dereplicated with the *derepFastq* function. Subsequently, the error rate was estimated from a subset of 1,000,000 reads for each run separately using the *learnErrors* function. These error rate estimates were used in the actual denoising function, *dada*, run with the option pool = T. The denoised forward and reverse reads were merged with *mergePairs* command in order to obtain amplicon sequence variants (ASVs). Finally, we created input files for further processing in *mothur*^55^: a fasta file and a group file (assigning sequences to samples). In summary, 83% (12 800 410/15 398 557) of raw reads passed quality control and denoising steps (Supplementary Table 9). We removed 11 out of 250 samples due to low number of reads (<1500). The remaining 239 (141 fecal and 98 saliva) samples contained on average 53 540 reads (±34 540).

We used *mothu*r to assign taxonomy, remove non-bacterial sequences and cluster OTUs, following the MiSeq standard operating procedure^60^. Briefly, we classified sequences with *mothur* implementation of RDP classifier^61^ using the RDP Release 11 taxonomy^62^ with 80% confidence cutoff and filtered out unclassified and non-bacterial sequences. We aligned the sequences to the V4 region of the SILVA v132 reference alignment^63^ and removed the chimeras with CATCh^64^. We used OptiClust^65^ with Matthews correlation coefficient (MCC) optimization to create 85–99%ID OTUs at 0.01 steps and applied 80% confidence cutoff for the consensus OTU taxonomy. OTU representative sequences were defined as the sequences with the smallest average distance to all the other sequences within a given OTU.

In order to explore the effect of OTU clustering method, we used also another, phylogeny-based approach, *bdtt*^66^, which creates the OTUs by slicing of a phylogenetic tree (whose construction is described below) at a specified evolutionary distance. We sliced the tree in the range 0.01–0.15 branch length at 0.01 steps. We modified the original *bdtt* script in order to add the new, sliced tree and the OTU table to the output. The consensus taxonomy was found as the lowest common ancestor of all sequences within an OTU.

The number of reads and ASVs per sample before and after quality control are reported in Supplementary Table 9. The number of reads and OTUs before and after filtering for each combination of clustering method and cutoff are listed in Supplementary Table 10. We identified in total 4,078 ASVs (grouped into 246 genera), 2,977 of which were found in the fecal microbiome (209 genera) and 2,025 in the saliva microbiome (198 genera).

#### Phylogenetic tree

The phylogenetic tree was created with RaxML^67^ following the protocol described in Stamatakis^68^. We imposed the monophyly of the classes within *Proteobacteria* on the tree topology as described in Groussin *et al*.^66^. We ran 100 independent fast tree searches with GTRGAMMA model, performed a bootstrap analysis (with autoMRE option, which automatically determines how many replicates are needed to get stable support values, in this case 550 trees) on the best tree and created a MR consensus tree. All independent trees were unique and the tree diversity was high (average Robinson-Foulds topology distance = 0.5029), indicating an unstable topology. This was further supported by low relative tree certainty from the bootstrap analysis (0.150089). Still, the best tree was significantly better than the suboptimal ones according to the Shimodaira-Hasegawa test^69^. Although the results of phylofactorization differed somewhat depending on the tree (not shown), they were overall congruent, so we used the best tree in all analyses (phylogenetic diversity, bdtt OTU clustering and phylofactorization).

#### Alpha diversity estimates

We calculated rarefactions curves for Good’s coverage and non-phylogenetic alpha diversity indices (observed species, Shannon’S H, inverse Simpson and Berger-Parker dominance) with 1000 iterations at 1000 sequence steps in *mothur*^55^. Based on the rarefaction results (not shown), we chose a subsampling depth of 6500 reads per sample for calculating the average (200 replicates) of the above alpha diversity indices. We then transformed these values into the effective number of species as described in Jost^70^. Effective number of species is the number of species in a perfectly even community that results in a given value of a diversity index. Diversity indices within this framework are mathematically defined as Hill numbers of different order (q) and can be linked to the more traditionally used richness and dominance/evenness indices. The order, q, defines how much weight is given to the abundant species: q = 0 ignores the abundance altogether (and is equal to species richness), q = 1 weighs the species exactly according to their abundance (and corresponds to exp(Shannon’s H)), q = 2 is the same as inverse Simpson index and gives emphasis to dominant species, whereas q = Inf corresponds to the inverse of Berger-Parker dominance, and thus depends only on the relative abundance of the most abundant species. The advantage of these indices is that they are intuitive and easily interpretable: they have common units and possess doubling property (i.e. diversity of a union of two communities with completely distinct species is the sum of diversities of the two component communities). Jost^70^ refers to these diversity indices as “diversity of order 0, 1, 2”. We use here their mathematical denotations - ^0^D, ^1^D, ^2^D and ^Inf^D - for brevity.

Chao *et al*.^71^ developed a corresponding Hill-number framework for phylogenetic diversity. The main idea is the same, but here the diversity is defined as effective number of lineages, corresponding to the phylogenetic diversity of a perfectly even community composed of perfectly distinct species. We used the *hillR* R package^72^ to calculate average phylogenetic diversity indices of order 0, 1 and 2 of 20 dataset replicates subsampled to 6500 reads/sample. We refer to these indices as ^0^D_ph_, ^1^D_ph_, ^2^D_ph._.

#### Filtering for beta diversity analysis and community type determination

In order to get rid of the noise and to reduce the complexity of the dataset, we performed a two-step unsupervised filtering implemented in the *PERFect* package^73^. Briefly, the method compares the total covariance of the community data (“OTU table”) before and after removal of a taxon. This “difference in filtering loss” is then compared with a null distribution and the taxon is retained or discarded for a given significance level alpha (here alpha = 0.1). Based on the empirical evidence and authors’ suggestions, we assumed that at least 50% of taxa were not informative and therefore fitted a skew-normal distribution to the observed filtering loss values. In the first step, called simultaneous filtering, the taxa are ordered according to the number of occurrences and filtered sequentially as long as the filtering loss values come from the null distribution; in the second step, called permutation filtering, the taxa are ordered according to the p-values from the first step and filtered based on an additional permutation step (1000 replicates) that ensures that only the taxa that are uninformative for any combination of retained taxa are removed. We filtered the gut and saliva microbiome datasets separately. Filtering for beta diversity analyses resulted in 282 ASVs for the fecal and 110 ASVs for the saliva dataset (Supplementary Tables 9 and 10). This filtering step reduced the number of reads by 31% and 9% in the fecal and saliva datasets respectively, while eliminating on average 90% and 88% of the ASVs/OTUs/genera, regardless of the clustering method and cutoff.

As the compositional methods that we used to statistically analyze the data do not allow zero values, we replaced zeros utilizing the Bayesian multiplicative replacement function (cmult.repl, method = “GBM”) implemented in the *zCompositions* R package^74^.

For distance-based analyses, we performed a centered log ratio (clr) transformation^75^ implemented in the *CoDaSeq* R package^76,77^ and calculated the Euclidean distances, corresponding to Aitchison distances of raw compositional data^78^. For community type determination, we grouped the non-filtered ASV dataset at the genus level with the *phyloseq* “tax_glom” command^79^.

#### Statistical analysis: microbiome diversity and composition along the urbanization gradient

To examine how the human microbiome diversity changes along the urbanization gradient, we analyzed the gut and saliva microbiome separately.

For alpha diversity, we fitted generalized least squares (gls) linear models with the package *nlme*^80^, with weights allowing different variances for each population in case of heteroscedasticity, i.e. if the Levene’s test of homogeneity of variance (*car* package^81^) was significant at alpha = 0.05.

To identify the taxa (ASVs and OTUs) whose relative abundances differed between the populations, we used the *ALDEx2* package^76,82^. We considered the taxa as differentially abundant only if Benjamini-Hochberg adjusted p-values of both Kruskal-Wallis and glm test were significant (p <= 0.05).

Finally, in order to identify the lineages that most predictably vary along the urbanization gradient regardless of the classification level, we performed a phylofactorization using the *phylofactor* package^83^, with a custom objective function (the same *gls* model as used in the alpha diversity analysis), F-statistic as a criterion for choosing the best edge, and KS test as the stopping function.

#### Statistical analysis: community types

In order to classify the microbiomes into the optimal number of categories, we performed Dirichlet-multinomial mixture (DMM) modelling^84^ on the genus-level data with the *DirichletMultinomial* package^85^. We chose the optimal number of components based on the Laplace goodness-of-fit of models with one to ten components. We calculated associations between enterotypes (gut community types^86^) and stomatotypes (saliva community types^46^), as well as between the community types and urbanization using Fisher’s exact test as in^44^.

#### Statistical analysis: factors associated with community diversity and composition

As food-frequency questionnaire (FFQ) data were not available for semi-urban samples, we performed each of the following analyses three times: for all samples without FFQ data, and for rural-urban samples with and without FFQ data.

The metadata that we collected included both numerical and categorical, often correlated variables (Supplementary Table 4 and Fig. 1). As it is often not trivial to decide which of the correlated factors are important (representing a problem for stepwise variable selection) and as we wanted to make as few assumptions about the data as possible (e.g. linearity), we used random forest classification and regression for the prediction of community types and alpha diversity, respectively. Random forest regression/classification identifies the best set of predictors, without assuming any particular relationship between the independent and response variables. We used conditional forest algorithm^87^ implemented in the *partykit* package^88^. We followed the guidelines from^48^ and calculated conditional variable importance with the *party* package and cutoff = 0.2^49^ in order to avoid biases due to different scale of predictors and correlations between them. We used the *caret* package^89^ to split the dataset into a training (80%) and test (20%) subsets and to find the best value for the “mtry” (i.e. available number of variables for splitting at each node) parameter. We used the minimization of RMSE (root mean square error) as the selection criterion for regression and the maximization of Cohen’s Kappa for classification. We ran ten independent iterations on each dataset to get realistic estimates of the mean and variability of the variable importance and the goodness of fit. As there is no clear cutoff for variable selection based on their importance, we applied several approaches. The most relaxed approach was the inclusion of random variables in the models: the factors with lower mean importance than the random variable with the highest value were considered unimportant. The second approach was to keep all the variables with a mean importance above the 95% quantile of all mean importance values. The final approach was to split the mean importances into two groups by kmeans clustering. In the last two approaches, we discarded all predictors if any of the random variables was in the “important” group. We used both variable importance and goodness-of-fit estimates (including R-squared and variance explained by the model). If the proportion of the variance explained or the model accuracy was low (R-squared < 0.1 or Kappa < 0.3), we did not interpret the results.

We analyzed potential associations between the community composition and the metadata by a distance-based approach inspired by Gloor *et al*.^90^ and Falony *et al*.^25^. Briefly, we performed a Principal Component Analysis on the equivalents of Aitchison distances with the package *vegan*^91^. We found the variables significantly associated with the first three axes of variation using the *envfit* function. If some of the significant variables were correlated (correlation coefficient >= 0.8, Supplementary Table 3), we selected the one that explained more variation in *envfit* and discarded the rest. We then used these selected variables as a scope for *ordistep* (bidirectional) variable selection. Finally, we identified the ASVs (or OTUs) whose coordinates were in the lower 2.5% and the upper 97.5% quantile on the first three PCA axes as well as those with the distances in the 2.5% and the upper 97.5% quantile from the coordinates of the selected environmental variables (calculated with the *cdist* function from the *rdist* package^92^.

#### Statistical analysis: differences between the gut and saliva microbiome

We investigated a potential correlation between the gut and saliva microbiome diversity within individual using Pearson’s r coefficient, with nonparametric bootstrap 95% confidence intervals calculated with the *boot* package^93,94^. In order to determine if the gut and saliva microbiome within an individual share more species than expected by chance, we compared the observed within-individual beta diversity values (both Bray-Curtis and species turnover measure that accounts for the differences in species richness between the samples; the measure varies between 1 for identical communities and 2 for completely distinct communities^95^) with the null distribution created by shuffling the sample labels 10 000 times. We either used free permutations or allowed permutations only within the population, to account for the differences between them. We calculated the p-values as observed rank/10 001 and the standardized effect size as (observed mean - random mean)/random standard deviation.

## Supplementary information

Supplementary information

Supplementary information2

## Data Availability

All full and filtered datasets used in our study are stored as *phyloseq*^79^ objects or tables and available on Figshare. The raw data are deposited in European Nucleotide Archive under the accession number PRJEB30836. Other datasets and alignments that were generated and used within the current study are available from the authors upon request.

## Code availability

All scripts used in the study are available from the authors upon request.

## References

[1] Yatsunenko, T. *et al*. Human gut microbiome viewed across age and geography. *Nature* 1–7, 10.1038/nature11053 (2012). 10.1038/nature11053 PMC3376388 22699611

[2] Schnorr, S. L. *et al*. Gut microbiome of the Hadza hunter-gatherers. *Nat. Commun*. **5** (2014). 10.1038/ncomms4654 PMC3996546 24736369

[3] Clemente JC (2015). The microbiome of uncontacted Amerindians. Sci. Adv..

[4] Martínez I (2015). The Gut Microbiota of Rural Papua New Guineans: Composition, Diversity Patterns, and Ecological Processes. Cell Rep..

[5] Obregon-Tito A (2015). Subsistence strategies in traditional societies distinguish gut microbiomes. Nat. Commun..

[6] Gomez A (2016). Gut Microbiome of Coexisting BaAka Pygmies and Bantu Reflects Gradients of Traditional Subsistence Patterns. Cell Rep..

[7] Vangay P (2018). US Immigration Westernizes the Human Gut Microbiome. Cell.

[8] Hansen MEB (2019). Population structure of human gut bacteria in a diverse cohort from rural Tanzania and Botswana. Genome Biol..

[9] Smits SA (2017). Seasonal cycling in the gut microbiome of the Hadza hunter-gatherers of Tanzania. Science.

[10] Mancabelli L (2017). Meta-analysis of the human gut microbiome from urbanized and pre-agricultural populations: The urbanization/industrialization of humans and gut microbiomes. Environ. Microbiol..

[11] Dominguez-Bello, M. G., Godoy-Vitorino, F., Knight, R. & Blaser, M. J. Role of the microbiome in human development. *Gut gutjn* l-2018-317503, 10.1136/gutjnl-2018-317503 (2019). 10.1136/gutjnl-2018-317503 PMC6580755 30670574

[12] Blaser MJ (2017). The theory of disappearing microbiota and the epidemics of chronic diseases. Nat. Rev. Immunol..

[13] Sonnenburg ED, Sonnenburg JL (2019). The ancestral and industrialized gut microbiota and implications for human health. Nat. Rev. Microbiol..

[14] Makki K, Deehan EC, Walter J, Bäckhed F (2018). The Impact of Dietary Fiber on Gut Microbiota in Host Health and Disease. Cell Host Microbe.

[15] Blaser MJ, Falkow S (2009). What are the consequences of the disappearing human microbiota?. Nat. Rev. Microbiol..

[16] Dikongué E, Ségurel L (2017). Latitude as a co-driver of human gut microbial diversity?. BioEssays.

[17] Jha, A. R. *et al*. Gut microbiome transition across a lifestyle gradient in Himalaya, 10.1101/253450 (2018). 10.1371/journal.pbio.2005396 PMC6237292 30439937

[18] Girard C, Tromas N, Amyot M, Shapiro BJ (2017). Gut Microbiome of the Canadian Arctic Inuit. mSphere.

[19] Tyakht AV (2013). Human gut microbiota community structures in urban and rural populations in Russia. Nat. Commun..

[20] Ayeni FA (2018). Infant and Adult Gut Microbiome and Metabolome in Rural Bassa and Urban Settlers from Nigeria. Cell Rep..

[21] Winglee, K. *et al*. Recent urbanization in China is correlated with a Westernized microbiome encoding increased virulence and antibiotic resistance genes. *Microbiome* **5** (2017). 10.1186/s40168-017-0338-7 PMC5603068 28915922

[22] Zhang J (2014). Mongolians core gut microbiota and its correlation with seasonal dietary changes. Sci. Rep..

[23] Das B (2018). Analysis of the Gut Microbiome of Rural and Urban Healthy Indians Living in Sea Level and High Altitude Areas. Sci. Rep..

[24] Stagaman K (2018). Market Integration Predicts Human Gut Microbiome Attributes across a Gradient of Economic Development. mSystems.

[25] Falony G (2016). Population-level analysis of gut microbiome variation. Science.

[26] Lokmer A (2019). Use of shotgun metagenomics for the identification of protozoa in the gut microbiota of healthy individuals from worldwide populations with various industrialization levels. PLOS ONE.

[27] Chabé, M., Lokmer, A. & Ségurel, L. Gut Protozoa: Friends or Foes of the Human Gut Microbiota? *Trends Parasitol*., 10.1016/j.pt.2017.08.005 (2017). 10.1016/j.pt.2017.08.005 28870496

[28] Morton ER (2015). Variation in rural African gut microbiota is strongly correlated with colonization by Entamoeba and subsistence. PLoS Genet.

[29] Andersen LO, Stensvold CR (2016). Blastocystis in Health and Disease: Are We Moving from a Clinical to a Public Health Perspective?. J. Clin. Microbiol..

[30] Audebert, C. *et al*. Colonization with the enteric protozoa Blastocystis is associated with increased diversity of human gut bacterial microbiota. *Sci*. *Rep*. **6** (2016). 10.1038/srep25255 PMC4857090 27147260

[31] Beghini F (2017). Large-scale comparative metagenomics of Blastocystis, a common member of the human gut microbiome. ISME J..

[32] Tito, R. Y. *et al*. Population-level analysis of Blastocystis subtype prevalence and variation in the human gut microbiota. *Gut* gutjnl-2018-316106, 10.1136/gutjnl-2018-316106 (2018). 10.1136/gutjnl-2018-316106 PMC6582744 30171064

[33] Lassalle, F. *et al*. Oral microbiomes from hunter-gatherers and traditional farmers reveal shifts in commensal balance and pathogen load linked to diet. *Mol*. *Ecol*., 10.1111/mec.14435 (2017). 10.1111/mec.14435 29165844

[34] Nasidze I (2011). High Diversity of the Saliva Microbiome in Batwa Pygmies. PLOS ONE.

[35] Li J (2014). Comparative analysis of the human saliva microbiome from different climate zones: Alaska, Germany, and Africa. BMC Microbiol..

[36] Ozga AT (2016). Oral microbiome diversity among Cheyenne and Arapaho individuals from Oklahoma. Am. J. Phys. Anthropol..

[37] Takeshita T (2014). Distinct composition of the oral indigenous microbiota in South Korean and Japanese adults. Sci. Rep..

[38] Si, J., Lee, C. & Ko, G. Oral Microbiota: Microbial Biomarkers of Metabolic Syndrome Independent of Host Genetic Factors. *Front. Cell. Infect. Microbiol*. **7** (2017). 10.3389/fcimb.2017.00516 PMC5736563 29326886

[39] De Filippo, C. *et al*. Diet, Environments, and Gut Microbiota. A Preliminary Investigation in Children Living in Rural and Urban Burkina Faso and Italy. *Front. Microbiol*. **8** (2017). 10.3389/fmicb.2017.01979 PMC5645538 29081768

[40] Shirley, D.-A. T., Farr, L., Watanabe, K. & Moonah, S. A Review of the Global Burden, New Diagnostics, and Current Therapeutics for Amebiasis. *Open Forum Infect. Dis*. **5** (2018). 10.1093/ofid/ofy161 PMC6055529 30046644

[41] Lukeš J, Stensvold C, Jirků-Pomajbíková K, Wegener Parfrey L (2015). Are Human Intestinal Eukaryotes Beneficial or Commensals?. PLoS Pathog..

[42] Vandeputte D (2016). Stool consistency is strongly associated with gut microbiota richness and composition, enterotypes and bacterial growth rates. Gut.

[43] Segata N (2012). Composition of the adult digestive tract bacterial microbiome based on seven mouth surfaces, tonsils, throat and stool samples. Genome Biol..

[44] Ding T, Schloss PD (2014). Dynamics and associations of microbial community types across the human body. Nature.

[45] Olsen, I. & Yamazaki, K. Can oral bacteria affect the microbiome of the gut? *J*. *Oral Microbiol*. **11** (2019). 10.1080/20002297.2019.1586422 PMC6427756 30911359

[46] Willis JR (2018). Citizen science charts two major “stomatotypes” in the oral microbiome of adolescents and reveals links with habits and drinking water composition. Microbiome.

[47] Lira-Junior R, Åkerman S, Klinge B, Boström EA, Gustafsson A (2018). Salivary microbial profiles in relation to age, periodontal, and systemic diseases. PLOS ONE.

[48] Strobl C, Boulesteix A-L, Zeileis A, Hothorn T (2007). Bias in random forest variable importance measures: Illustrations, sources and a solution. BMC Bioinformatics.

[49] Strobl C, Boulesteix A-L, Kneib T, Augustin T, Zeileis A (2008). Conditional variable importance for random forests. BMC Bioinformatics.

[50] Sharma S (1996). Development of food frequency questionnaires in three population samples of African origin from Cameroon, Jamaica and Caribbean migrants to the UK. Eur. J. Clin. Nutr..

[51] Lewis SJ, Heaton KW (1997). Stool Form Scale as a Useful Guide to Intestinal Transit Time. Scand. J. Gastroenterol..

[52] Quinque D, Kittler R, Kayser M, Stoneking M, Nasidze I (2006). Evaluation of saliva as a source of human DNA for population and association studies. Anal. Biochem..

[53] Poyet, M. *et al*. A library of human gut bacterial isolates paired with longitudinal multiomics data enables mechanistic microbiome research. *Nat*. *Med*., 10.1038/s41591-019-0559-3 (2019). 10.1038/s41591-019-0559-3 31477907

[54] R Core Team. R: A Language and Environment for Statistical Computing. (R Foundation for Statistical Computing, 2019).

[55] Schloss PD (2009). Introducing mothur: Open-Source, Platform-Independent, Community-Supported Software for Describing and Comparing Microbial Communities. Appl. Environ. Microbiol..

[56] Stekhoven DJ, Bühlmann P (2012). MissForest—non-parametric missing value imputation for mixed-type data. Bioinformatics.

[57] Mangiafico, S. *rcompanion: Functions to Support Extension Education Program Evaluation* (2019).

[58] Lê S, Josse J, Husson F (2008). FactoMineR: An R Package for Multivariate Analysis. J. Stat. Softw..

[59] Callahan BJ (2016). DADA2: High-resolution sample inference from Illumina amplicon data. Nat. Methods.

[60] Kozich J, Westcott S, Baxter N, Highlander S, Schloss P (2013). Development of a Dual-Index Sequencing Strategy and Curation Pipeline for Analyzing Amplicon Sequence Data on the MiSeq Illumina Sequencing Platform. Appl. Environ. Microbiol..

[61] Wang Q, Garrity GM, Tiedje JM, Cole JR (2007). Naive Bayesian Classifier for Rapid Assignment of rRNA Sequences into the New Bacterial Taxonomy. Appl. Environ. Microbiol..

[62] Cole JR (2014). Ribosomal Database Project: data and tools for high throughput rRNA analysis. Nucleic Acids Res..

[63] Gl?ckner, F. O. *et al*. 25 years of serving the community with ribosomal RNA gene reference databases and tools. *J*. *Biotechnol*., 10.1016/j.jbiotec.2017.06.1198 (2017). 10.1016/j.jbiotec.2017.06.1198 28648396

[64] Mysara M, Saeys Y, Leys N, Raes J, Monsieurs P (2015). CATCh, an Ensemble Classifier for Chimera Detection in 16S rRNA Sequencing Studies. Appl. Environ. Microbiol..

[65] Westcott SL, Schloss PD (2017). OptiClust, an Improved Method for Assigning Amplicon-Based Sequence Data to Operational Taxonomic Units. mSphere.

[66] Groussin M (2017). Unraveling the processes shaping mammalian gut microbiomes over evolutionary time. Nat. Commun..

[67] Stamatakis A (2014). RAxML version 8: a tool for phylogenetic analysis and post-analysis of large phylogenies. Bioinformatics.

[68] Stamatakis A (2015). Using RAxML to Infer Phylogenies. Curr. Protoc. Bioinforma..

[69] Shimodaira H, Hasegawa M (1999). Multiple Comparisons of Log-Likelihoods with Applications to Phylogenetic Inference. Mol. Biol. Evol..

[70] Jost L (2006). Entropy and diversity. Oikos.

[71] Chao A, Chiu C-H, Jost L (2010). Phylogenetic diversity measures based on Hill numbers. Philos. Trans. R. Soc. B. Biol. Sci..

[72] Li D (2018). hillR: taxonomic, functional, and phylogenetic diversity and similarity through Hill Numbers. J. Open Source Softw..

[73] Smirnova, E., Huzurbazar, S. & Jafari, F. PERFect: PERmutation Filtering test for microbiome data. Biostatistics, 10.1093/biostatistics/kxy020. 10.1093/biostatistics/kxy020 PMC6797060 29917060

[74] Palarea-Albaladejo J, Martin-Fernandez JA (2015). zCompositions – R package for multivariate imputation of left-censored data under a compositional approach. Chemom. Intell. Lab. Syst..

[75] Aitchison J (1982). The statistical analysis of compositional data. J. R. Stat. Soc. Ser. B Methodol..

[76] Gloor GB, Macklaim JM, Fernandes AD (2016). Displaying Variation in Large Datasets: Plotting a Visual Summary of Effect Sizes. J. Comput. Graph. Stat..

[77] Gloor, G. B., Macklaim, J. M., Pawlowsky-Glahn, V. & Egozcue, J. J. Microbiome Datasets Are Compositional: And This Is Not Optional. *Front*. *Microbiol*. **8** (2017). 10.3389/fmicb.2017.02224 PMC5695134 29187837

[78] Aitchison J (1992). On criteria for measures of compositional difference. Math. Geol..

[79] McMurdie PJ, Holmes S (2013). phyloseq: An R Package for Reproducible Interactive Analysis and Graphics of Microbiome Census Data. PLOS ONE.

[80] Pinheiro, J., Bates, D., DebRoy, S., Sarkar, D. & R Core Team. *nlme: Linear and Nonlinear Mixed Effects Models* (2019).

[81] Fox, J. & Weisberg, S. An R *Companion to Applied Regression*. (Sage, 2019).

[82] Fernandes AD (2014). Unifying the analysis of high-throughput sequencing datasets: characterizing RNA-seq, 16S rRNA gene sequencing and selective growth experiments by compositional data analysis. Microbiome.

[83] Washburne, A. D. *et al*. Phylofactorization: a graph partitioning algorithm to identify phylogenetic scales of ecological data. *Ecol*. *Monogr*, e01353, 10.1002/ecm.1353 (2019).

[84] Holmes I, Harris K, Quince C (2012). Dirichlet Multinomial Mixtures: Generative Models for Microbial Metagenomics. PLOS ONE.

[85] Morgan, M. DirichletMultinomial: Dirichlet-Multinomial Mixture Model Machine Learning for Microbiome Data (2019).

[86] Arumugam M (2011). Enterotypes of the human gut microbiome. Nature.

[87] Hothorn T, Hornik K, Zeileis A (2006). Unbiased Recursive Partitioning: A Conditional Inference Framework. J. Comput. Graph. Stat..

[88] Hothorn T, Zeileis A (2015). partykit: A Modular Toolkit for Recursive Partytioning in R. J. Mach. Learn. Res..

[89] Kuhn, M. *The caret Package* (2009).

[90] Gloor GB, Reid G (2016). Compositional analysis: a valid approach to analyze microbiome high-throughput sequencing data. Can. J. Microbiol..

[91] Oksanen, J. *et al*. vegan: Community Ecology Package (2019).

[92] Blaser, N. rdist: Calculate Pairwise Distances (2018).

[93] Davison, A. C. & Hinkley, D. V. *Bootstrap Methods and Their Applications*. (Cambridge University Press, 1997).

[94] Canty, A. & Ripley, B. D. *boot: Bootstrap R (S-Plus) Functions* (2019).

[95] Jost L (2007). Partitioning diversity into independent alpha and beta components. Ecology.

[96] OpenStreetMap contributors. Planet dump retrieved from, https://planet.osm.org. Available under the Open Database Licence, https://www.openstreetmap.org/copyright (2017).

