# Supplementary material for: Response of the human gut and saliva microbiome to urbanization in Cameroon
Source: Sci Rep. 2020 Feb 18;10:2856. doi: 10.1038/s41598-020-59849-9 (PMC7028744; doi:10.1038/s41598-020-59849-9)
Supplement: Supplementary file 1 — Supplementary information [file 41598_2020_59849_MOESM1_ESM.pdf]

Response of the human gut and saliva microbiome to urbanization in Cameroon

Ana Lokmer[1], Sophie Aflalo[1], Norbert Amougou[1], Sophie Lafosse[1], Alain Froment[1], Francis Ekwin Tabe[2], Mathilde Poyet[3,4], Mathieu Groussin[3,4], Rihlat Said-Mohamed[5], Laure Ségurel[1*]

[1] UMR7206 Eco-anthropologie, CNRS - MNHN - Univ Paris Diderot - Sorbonne Paris Cité, Paris, France

[2] Faculté de Médécine et des Sciences Biomédicales - Université Yaoundé 1, Cameroun

[3] Department of Biological Engineering / Center for Microbiome Informatics and Therapeutics, Massachusetts Institute of Technology, Cambridge, MA, USA

[4]The Broad Institute of MIT and Harvard, Cambridge, MA, USA

[5] SAMRC/WITS Developmental Pathways for Health Research Unit, Department of Paediatrics, School of Clinical Medicine, Faculty of Health Sciences,
University of the Witwatersrand, South Africa

Correspondence: laure.segurel@mnhn.fr, ana.lokmer@mnhn.fr

Additional results and discussion (community types, phylofactorization and OTU clustering)

Community types

(Supplementary Fig. 7 & Supplementary Fig. 8)

Microbiomes can be grouped into types based on their structure [1], which can simplify the analysis of the microbiome composition. We applied an approach based on Dirichlet-multinomial mixture (DMM) modelling[2] following Ding & Schloss[3] to define enterotypes (gut community types[1]) and stomatotypes (saliva community types[4]) in the studied populations (see Methods for details). In addition, in order to find out which factors best predict enterotypes and stomatotypes, we ran random forest classification models for each of the three data subsets (whole dataset, and rural-urban dataset with and without FFQ data) separately. We interpreted only the models with Kappa values (accuracy corrected for the chance agreement) > 0.3.
We identified four enterotypes and two stomatotypes, discussed in detail below. In summary, we found community types resembling those identified by other researchers in different populations around the globe, as well as the enterotypes and stomatotypes unique to our study. Unlike Ding & Schloss[3], we did not find any evidence of correlation between the gut and saliva community types.

Enterotype and stomatotype distribution in the studied populations.

	Enterotypes				Stomatotypes	
	F1	F2	F3	F4	S1	S2
Rural	26	9	32	11	20	22
Semi-urban	11	12	4	4	14	11
Urban	5	19	0	8	26	5

Enterotypes

Dirichlet Multinomial Modelling (DMM) of the genus-agglomerated abundance data identified four enterotypes: F1, dominated by *Prevotella*; F2, enriched in *Ruminococcaceae*, unclassified *Clostridiales*, *Lachnospiraceae* and another unclassified bacterial genus; F3, which could be considered as a transient type between the first two; and F4, dominated by *Bacteroides* and *Lachnospiraceae*. All three populations differed significantly in enterotype distribution from each other (Fisher's exact test < 0.05), with 74% of rural individuals characterized by enterotypes F1 and F3, 74% of semi-urban individuals by enterotypes F1 and F2, and 59% of urban individuals by the enterotype F2. We found no urban individuals with

the F3 enterotype. In addition, the urban population had almost twice more individuals with F4 (25%) compared to the semi-urban (13%) and rural population (14%).

For all three data subsets, enterotypes were best predicted by the presence of *Entamoeba* sp., which was found in 60% of F2 and 75% of F3 individuals, but only in 28% of F1 and 9% of F4 individuals. The next most important factors were the water source and urbanization level, further followed by the Bristol stool scale score for the whole dataset only, with the highest-scoring individuals hosting mostly F1 and the lowest-scoring ones hosting F2, while F3 and F4 individuals were characterized by intermediate values.

The high prevalence of *Prevotella*-dominated enterotypes (F1 and F3) is not surprising for a country characterized by low level of industrialization, but it is interesting that some previous studies did not identify the *Ruminococcaceae* enterotype in lowly industrialized African populations[5,6], which was common in our study. On the other hand, Mobeen et al.[6] reported a *Ruminococcaceae*-dominated enterotype as typical for urban Colombians. Finally, whereas the *Bacteroides* enterotype is expected to increase in prevalence with increasing urbanization and industrialization[5–8], it is surprising that we found no direct association with diet, despite the dietary differences between rural and urban populations and the previously established links with diet[7]. Still, it is possible that this is due to the fact that no single dietary item, but rather an average diet is associated with the enterotype variation and is thus in our case best predicted by the urbanization level as a whole. Finally, similar to Vandeputte et al.[9] and Falony et al.[10] who studied industrialized populations, we observed an association between the Bristol stool scale score and enterotypes, with *Ruminococcaceae* scoring lowest and *Prevotella* enterotype highest on the scale. In conclusion, the observed discrepancies with the previous studies could partially be explained by the scarcity of data on enterotypes in Africa[11], taken that the identity and prevalence of enterotypes may vary considerably with geography[5,6,11,12].

Stomatotypes

We identified two stomatotypes in the studied populations: S1, dominated by *Prevotella*, *Streptococcus* and to lesser extent by *Veillonella*, *Neisseria* and Pasteurellaceae, and S2, enriched in an *Enterobacteriaceae* genus. 84% of individuals in urban population hosted the S1 stomatotype, which was significantly different from the semi-urban and rural populations (Fisher's exact test, p=0.005 and 0.054), where both stomatotypes were equally represented.

Regarding the best stomatotype predictors, only the rural-urban data subsets had Kappa values > 0.3 (representing a "fair agreement"[13]) and were considered for interpretation. In both cases, the stomatotype was best predicted by habitat-related factors, and additionally with the consumption of peanuts if FFQ data were included. Specifically, S2 individuals were more likely to live in houses without cement floor and tap water, to have animals in household and eat more peanuts, all of which were associated with the rural environment.

Stomatotypes[4] have received much less attention than enterotypes so far. Ding & Schloss[3] identified four different saliva bacterial community types, whereas Willis et al.[4] found two. Our S2, dominated by *Prevotella*, overall corresponds to Ding's & Schloss's[3] A and C and Willis's et al. [4] stomatotype 2. On the other hand, *Enterobacteriaceae* (enriched in our S1) have been previously found in significant concentrations in the saliva microbiome of African populations[14–16], whereas they are virtually absent in other parts of the world[3,4,14,16,17]). Li et al.[16] proposed that high abundance of *Enterobacteriaceae* could be due to high temperatures, but this does not explain the absence of *Enterobacteriaceae* in hunter-gatherer and traditional agriculturist populations from Phillipines[17]. Whereas Ding & Schloss[3] found significant association between the *Prevotella* dominated stomatotypes and enterotypes, we found no such correlation. However, it is important to mention that *Prevotella* was abundant in both stomatoypes defined here, despite the *Enterobacteriacae* dominance in S1.

Finally, it is noteworthy that grouping of the saliva microbiomes into three stomatotypes was only slightly worse according to the goodness-of-fit statistics. This third stomatotype was dominated by *Streptococcus*, followed by *Prevotella*, *Neisseria* and unclassified *Pasteurellaceae*. This stomatoype shared some characteristics with the S1 of Willis et al.[4] (higher relative abundance of *Neisseria* and to certain extent *Haemophilus*) and was mostly dominant in the urban population.

Phylofactorization

(Supplementary Table 6, Supplementary Fig. 5, two figures below)

Although ASV (amplicon sequence variants) seem to be best suited for assessing fine ecological differences between similar ecosystems[18,19], different factors may act at different scales (e.g. [20]) and clustering may be a better approach in some cases[18]. However, it is often difficult to know *a priori* which scales are important and, in addition, multiple scales may be affected by the factor(s) of interest. In order to address this issue, Washburne et al.[21] developed a method called phylofactorization (implemented in the R package *phylofactor*), based on a graph-partitioning algorithm that cuts the phylogenetic tree into groups of lineages ("phylofactors") whose relative abundance changes the most in response to the studied factor, in our case to urbanization.

Briefly, phylofactors are created based on some objective function. We used a custom function - a generalized least squares model as implemented in the function *gls* in the R package *nlme*[22], with variance structure specified if needed (see Methods for the alpha diversity analysis. We used the maximization of F-statistic as a criterion for phylofactor selection. According to the authors[21], in this way the selected phylofactors represent the lineages that most predictably vary with the studied factor. We did not pre-specify the number of phylofactors, but used a KS (Kolmogorov-Smirnov) test as a stopping criterion as described in [21].

Phylofactors in the gut microbiome

Phylofactorization detected 104 lineages (ASVs or clades) consistently affected by urbanization in the gut microbiome (based on a tree with 561 edges). The fact that only a minority (17) of these phylofactors encompassed more than a single ASV indicates that urbanization in this case affected the gut microbiome mostly at a fine phylogenetic scale. The results were overall concordant with ALDex2 (see Results and Supplementary Table 5), although the relative importance and statistical significance differed to certain extent between the methods. For example, the most differentially abundant OTU (*Succinivibrio*) according to ALDEx2 was selected only as the 17th phylofactor (*Aeromonadales*). However, the direction of changes and qualitative conclusions remained concordant.

Interestingly, 16 phylofactors were composed of one or more *Prevotella* ASVs that all had consistently higher abundance in rural samples, suggesting that different strains disappear at different rates in response to urbanization. *Prevotella* is often found in high relative abundance in rural populations[23] and it has been associated with a high-fiber[24] and high-carbohydrate diet[7]. On the other hand, Zhernakova et al.[25] found no association between *Prevotella* and carbohydrate intake and Das et al.[26] actually observed higher relative abundance of *Prevotella* in non-vegetarians compared to vegetarians in India. Furthermore, different *Prevotella* oligotypes have been linked with variation in dietary habits[27]. Therefore, we agree that caution is needed when interpreting genus-level changes[28], as these may conceal ecologically significant species/strain-level variation.

Apart from *Prevotella* phylofactors, we also observed opposed responses of various *Lachnospiraceae* and *Ruminococcaceae* to urbanization, corroborating the results obtained by ALDEx2. The taxonomy within these two families is not well defined[1], but the conflicting reports about their response to different factors suggest that they are indeed ecologically diversified. For example, Menni et al.[29] reported an increase in relative abundance of *Ruminococcaceae* and *Lachnospiraceae* associated with the fiber intake in humans, while Okeke et al.[30] found an increase in *Ruminococcacae* in the mice on a high-fat diet. In addition, *Lachnospiraceae* can mediate anti-inflammatory protection of helminth infection in mice[31]. On the other hand, Holm et al.[32] reported that worm-infected mice had microbiota with a lower relative abundance of bacterial genera from *Ruminococcaceae* and *Lachnospiraceae*. In our study, different *Lachnospiraceae* were associated with factors as different as the urbanization level, alcohol consumption or gut eukaryotes (Additional File 1: Table S7). Similarly, different *Ruminococcacae* primarily responded differently to the urbanization level, but some could be to certain extent associated with the presence of *Entamoeba* (Supplementary Table 7). Notably, the first phylofactor was composed of a *Ruminococcaceae* ASV whose abundance decreased with urbanization.

The largest phylofactor, F48, contained ten bacteria mostly classified as *Erysipelotrichaceae*, with relative abundances decreasing along the urbanization gradient. Interestingly, *Erysipelotrichaceae* have been linked with high-fat diet[33] and obesity[34], whereas their link with inflammatory bowel diseases is less clear (for review see[35]).

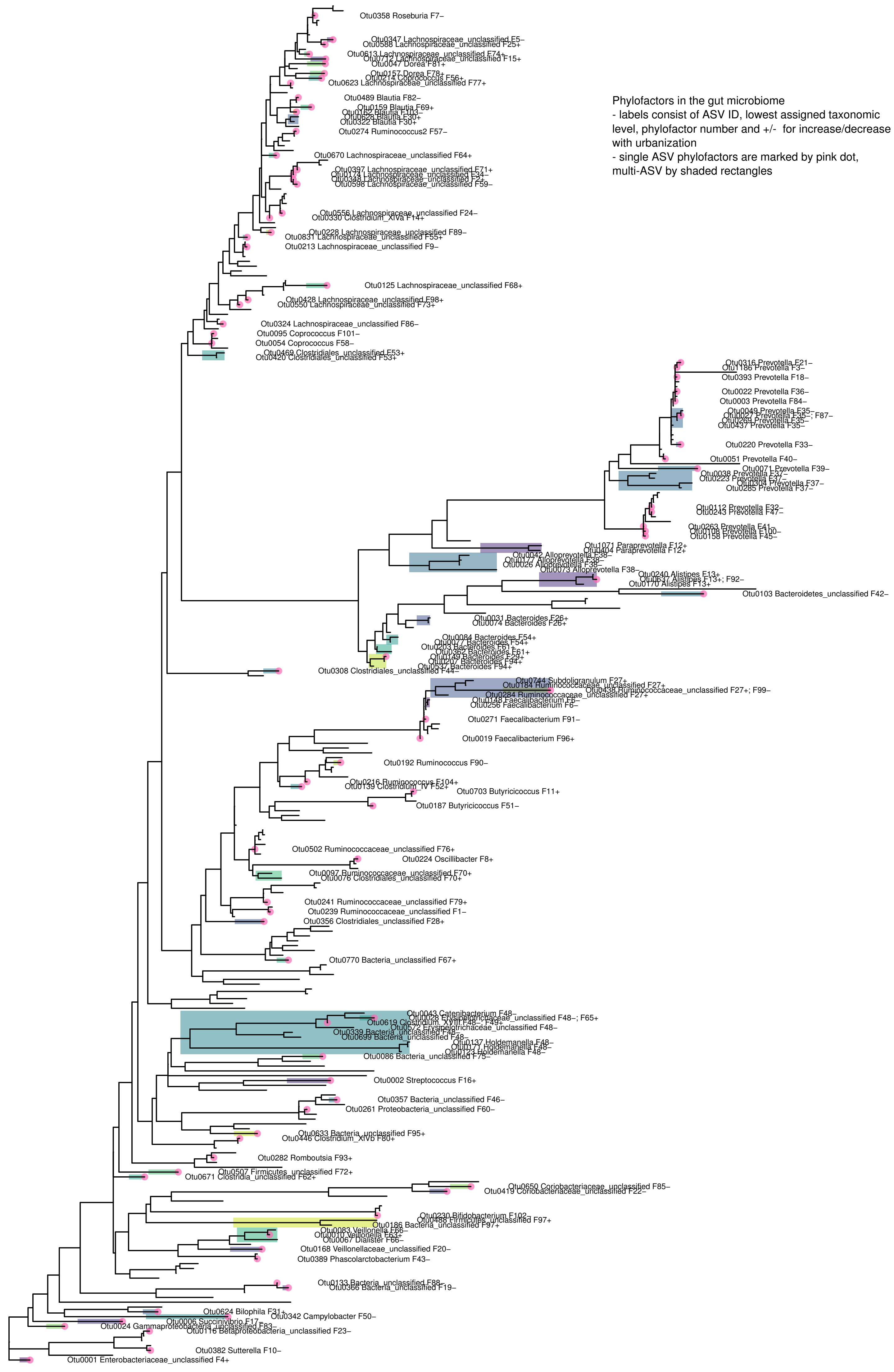
Phylofactors in the gut microbiome
- labels consist of ASV ID, lowest assigned taxonomic level, phylofactor number and +/- for increase/decrease with urbanization
- single ASV phylofactors are marked by pink dot, multi-ASV by shaded rectangles
Otu0358 Roseburia F7−
Otu0347 Lachnospiraceae_unclassified F5−
Otu0588 Lachnospiraceae_unclassified F25+
Otu0613 Lachnospiraceae_unclassified F74+
Otu0712 Lachnospiraceae_unclassified F15+
Otu0047 Dorea F81+
Otu0157 Dorea F78+
Otu0214 Coprococcus F56+
Otu0623 Lachnospiraceae_unclassified F77+
Otu0489 Blautia F82−
Otu0159 Blautia F69+
Otu0162 Blautia F103−
Otu0628 Blautia F30+
Otu0322 Blautia F30+
Otu0274 Ruminococcus2 F57−
Otu0670 Lachnospiraceae_unclassified F64+
Otu0397 Lachnospiraceae_unclassified F71+
Otu0174 Lachnospiraceae_unclassified F34−
Otu0348 Lachnospiraceae_unclassified F2+
Otu0598 Lachnospiraceae_unclassified F59−
Otu0556 Lachnospiraceae_unclassified F24−
Otu0330 Clostridium_XlVa F14+
Otu0228 Lachnospiraceae_unclassified F89−
Otu0831 Lachnospiraceae_unclassified F55+
Otu0213 Lachnospiraceae_unclassified F9−
Otu0125 Lachnospiraceae_unclassified F68+
Otu0428 Lachnospiraceae_unclassified F98+
Otu0550 Lachnospiraceae_unclassified F73+
Otu0324 Lachnospiraceae_unclassified F86−
Otu0095 Coprococcus F101−
Otu0054 Coprococcus F58−
Otu0469 Clostridiales_unclassified F53+
Otu0420 Clostridiales_unclassified F53+
Otu0316 Prevotella F21−
Otu1186 Prevotella F3−
Otu0393 Prevotella F18−
Otu0022 Prevotella F36−
Otu0003 Prevotella F84−
Otu0049 Prevotella F35−
Otu0027 Prevotella F35−; F87−
Otu0263 Prevotella F35−
Otu0437 Prevotella F35−
Otu0220 Prevotella F33−
Otu0051 Prevotella F40−
Otu0071 Prevotella F39−
Otu0038 Prevotella F37−
Otu0223 Prevotella F37−
Otu1304 Prevotella F37−
Otu0285 Prevotella F37−
Otu0112 Prevotella F32−
Otu0243 Prevotella F47−
Otu0263 Prevotella F41−
Otu0106 Prevotella F100−
Otu0158 Prevotella F45−
Otu1071 Paraprevotella F12+
Otu0404 Paraprevotella F12+
Otu0042 Alloprevotella F38−
Otu0177 Alloprevotella F38−
Otu0026 Alloprevotella F38−
Otu0073 Alloprevotella F38−
Otu0240 Alistipes F13+
Otu0637 Alistipes F13+; F92−
Otu0170 Alistipes F13+
Otu0103 Bacteroidetes_unclassified F42−
Otu0031 Bacteroides F26+
Otu0074 Bacteroides F26+
Otu0084 Bacteroides F54+
Otu0077 Bacteroides F54+
Otu0203 Bacteroides F61+
Otu0362 Bacteroides F61+
Otu0149 Bacteroides F29+
Otu0207 Bacteroides F94+
Otu0537 Bacteroides F94+
Otu0308 Clostridiales_unclassified F44−
Otu0744 Subdoligranulum F27+
Otu0184 Ruminococcaceae_unclassified F27+
Otu0438 Ruminococcaceae_unclassified F27+; F99−
Otu0284 Ruminococcaceae_unclassified F27+
Otu0148 Faecalibacterium F6−
Otu0256 Faecalibacterium F6−
Otu0271 Faecalibacterium F91−
Otu0019 Faecalibacterium F96+
Otu0192 Ruminococcus F90−
Otu0216 Ruminococcus F104+
Otu0139 Clostridium_IV F52+
Otu0703 Butyricicoccus F11+
Otu0187 Butyricicoccus F51−
Otu0502 Ruminococcaceae_unclassified F76+
Otu0224 Oscillibacter F8+
Otu0097 Ruminococcaceae_unclassified F70+
Otu0076 Clostridiales_unclassified F70+
Otu0241 Ruminococcaceae_unclassified F79+
Otu0239 Ruminococcaceae_unclassified F1−
Otu0356 Clostridiales_unclassified F28+
Otu0770 Bacteria_unclassified F67+
Otu0043 Catenibacterium F48−
Otu0028 Erysipelotrichaceae_unclassified F48−; F65+
Otu0619 Clostridium_XVIII F48−; F49+
Otu0572 Erysipelotrichaceae_unclassified F48−
Otu0339 Bacteria_unclassified F48−
Otu0699 Bacteria_unclassified F48−
Otu0137 Holdemanella F48−
Otu0171 Holdemanella F48−
Otu0123 Holdemanella F48−
Otu0086 Bacteria_unclassified F75−
Otu0002 Streptococcus F16+
Otu0357 Bacteria_unclassified F46−
Otu0261 Proteobacteria_unclassified F60−
Otu0633 Bacteria_unclassified F95+
Otu0446 Clostridium_XlVb F80+
Otu0282 Romboutsia F93+
Otu0507 Firmicutes_unclassified F72+
Otu0671 Clostridia_unclassified F62+
Otu0650 Coriobacteriaceae_unclassified F85−
Otu0419 Coriobacteriaceae_unclassified F22−
Otu0230 Bifidobacterium F102−
Otu0488 Firmicutes_unclassified F97+
Otu0186 Bacteria_unclassified F97+
Otu0083 Veillonella F66−
Otu0010 Veillonella F63+
Otu0067 Dialister F66−
Otu0168 Veillonellaceae_unclassified F20−
Otu0389 Phascolarctobacterium F43−
Otu0133 Bacteria_unclassified F88−
Otu0366 Bacteria_unclassified F19−
Otu0624 Bilophila F31+
Otu0342 Campylobacter F50−
Otu0006 Succinivibrio F17−
Otu0024 Gammaproteobacteria_unclassified F83−
Otu0116 Betaproteobacteria_unclassified F23−
Otu0382 Sutterella F10−
Otu0001 Enterobacteriaceae_unclassified F4+

Phylofactors in the saliva microbiome

For the saliva microbiome, 26 edges out of 217 tested significantly differentiated between the three populations, with nine phylofactors composed of more than one ASV. Unlike the bacteria in the gut microbiome, the response direction was generally consistent for most genera (e.g. *Streptococcus, Veilonella),* with an interesting exception of *Prevotella* (which always decreased with urbanization in the gut microbiome). Interestingly, the 20th phylofactor reflected the enrichment in *Enterobacteriaceae* the rural populations, which characterizes the stomatotype S1 discussed above. Due to this strong enrichment, relative abundances of the most phylofactors increase with increasing urbanization, with the exception of several *Prevotella* and *Pasteurellaceae* ASVs, both of which have been associated with various diseases[36], also with the healthy oral microbiomes of rural populations from non-industrialized countries[17].

Overall, phylofactorization offers a complementary approach to the methods with *a priori* defined taxonomic scales. However, its results depend on the quality of the phylogenetic tree[21] and high-quality trees will be needed in order to use its full potential. Still, it highlights the need to investigate the response of the gut microbiome to urbanization at the finest taxonomic scales in order to decipher ecological interactions within the gut ecosystem.

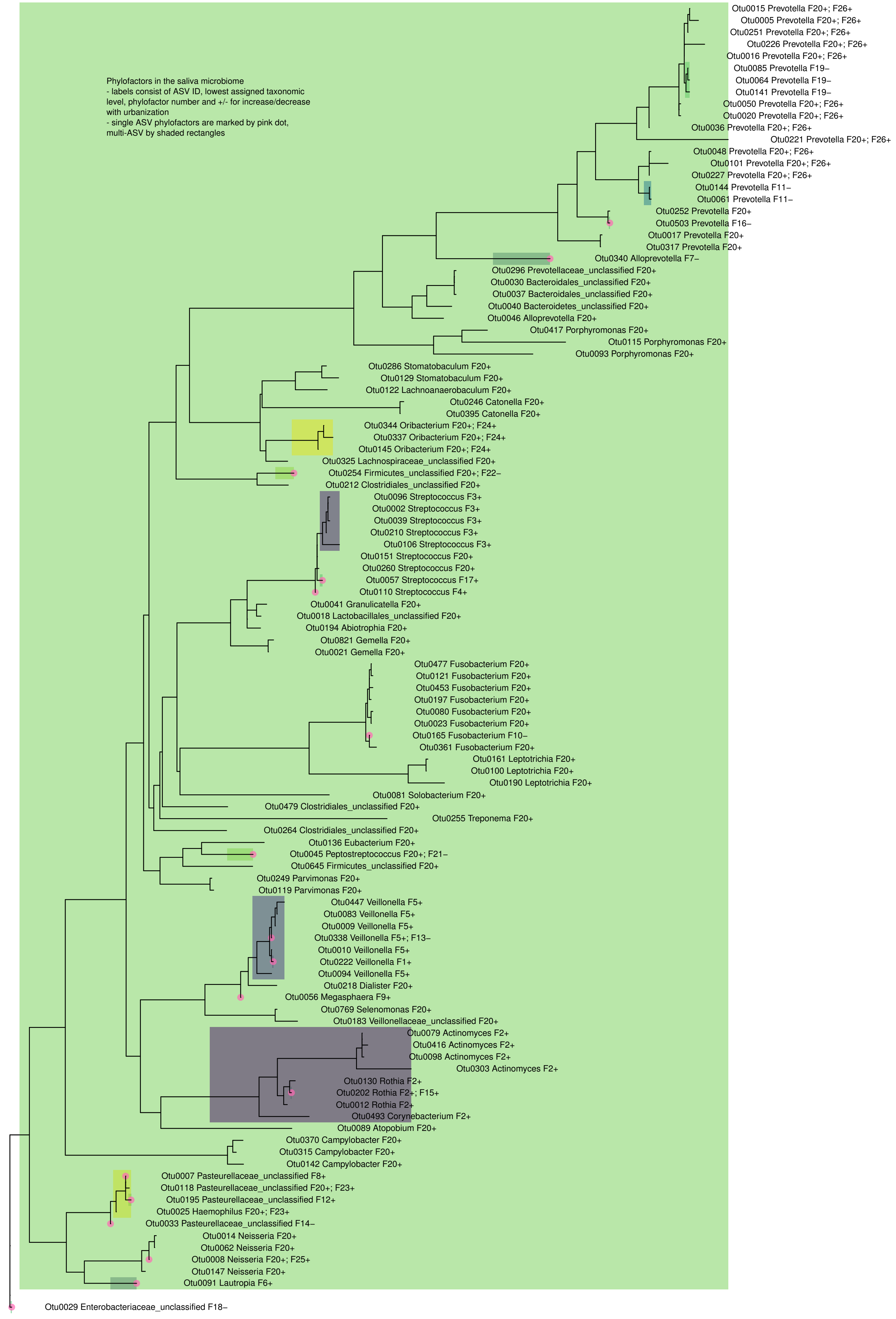

Phylofactors in the saliva microbiome
- labels consist of ASV ID, lowest assigned taxonomic level, phylofactor number and +/- for increase/decrease with urbanization
- single ASV phylofactors are marked by pink dot, multi-ASV by shaded rectangles
Otu0015 Prevotella F20+; F26+
Otu0005 Prevotella F20+; F26+
Otu0251 Prevotella F20+; F26+
Otu0226 Prevotella F20+; F26+
Otu0016 Prevotella F20+; F26+
Otu0085 Prevotella F19−
Otu0064 Prevotella F19−
Otu0141 Prevotella F19−
Otu0050 Prevotella F20+; F26+
Otu0020 Prevotella F20+; F26+
Otu0036 Prevotella F20+; F26+
Otu0221 Prevotella F20+; F26+
Otu0048 Prevotella F20+; F26+
Otu0101 Prevotella F20+; F26+
Otu0227 Prevotella F20+; F26+
Otu0144 Prevotella F11−
Otu0061 Prevotella F11−
Otu0252 Prevotella F20+
Otu0503 Prevotella F16−
Otu0017 Prevotella F20+
Otu0317 Prevotella F20+
Otu0340 Alloprevotella F7−
Otu0296 Prevotellaceae_unclassified F20+
Otu0030 Bacteroidales_unclassified F20+
Otu0037 Bacteroidales_unclassified F20+
Otu0040 Bacteroidetes_unclassified F20+
Otu0046 Alloprevotella F20+
Otu0417 Porphyromonas F20+
Otu0115 Porphyromonas F20+
Otu0093 Porphyromonas F20+
Otu0286 Stomatobaculum F20+
Otu0129 Stomatobaculum F20+
Otu0122 Lachnoanaerobaculum F20+
Otu0246 Catonella F20+
Otu0395 Catonella F20+
Otu0344 Oribacterium F20+; F24+
Otu0337 Oribacterium F20+; F24+
Otu0145 Oribacterium F20+; F24+
Otu0325 Lachnospiraceae_unclassified F20+
Otu0254 Firmicutes_unclassified F20+; F22−
Otu0212 Clostridiales_unclassified F20+
Otu0096 Streptococcus F3+
Otu0002 Streptococcus F3+
Otu0039 Streptococcus F3+
Otu0210 Streptococcus F3+
Otu0106 Streptococcus F3+
Otu0151 Streptococcus F20+
Otu0260 Streptococcus F20+
Otu0057 Streptococcus F17+
Otu0110 Streptococcus F4+
Otu0041 Granulicatella F20+
Otu0018 Lactobacillales_unclassified F20+
Otu0194 Abiotrophia F20+
Otu0821 Gemella F20+
Otu0021 Gemella F20+
Otu0477 Fusobacterium F20+
Otu0121 Fusobacterium F20+
Otu0453 Fusobacterium F20+
Otu0197 Fusobacterium F20+
Otu0080 Fusobacterium F20+
Otu0023 Fusobacterium F20+
Otu0165 Fusobacterium F10−
Otu0361 Fusobacterium F20+
Otu0161 Leptotrichia F20+
Otu0100 Leptotrichia F20+
Otu0190 Leptotrichia F20+
Otu0081 Solobacterium F20+
Otu0479 Clostridiales_unclassified F20+
Otu0255 Treponema F20+
Otu0264 Clostridiales_unclassified F20+
Otu0136 Eubacterium F20+
Otu0045 Peptostreptococcus F20+; F21−
Otu0645 Firmicutes_unclassified F20+
Otu0249 Parvimonas F20+
Otu0119 Parvimonas F20+
Otu0447 Veillonella F5+
Otu0083 Veillonella F5+
Otu0009 Veillonella F5+
Otu0338 Veillonella F5+; F13−
Otu0010 Veillonella F5+
Otu0222 Veillonella F1+
Otu0094 Veillonella F5+
Otu0218 Dialister F20+
Otu0056 Megasphaera F9+
Otu0769 Selenomonas F20+
Otu0183 Veillonellaceae_unclassified F20+
Otu0079 Actinomyces F2+
Otu0416 Actinomyces F2+
Otu0098 Actinomyces F2+
Otu0303 Actinomyces F2+
Otu0130 Rothia F2+
Otu0202 Rothia F2+; F15+
Otu0012 Rothia F2+
Otu0493 Corynebacterium F2+
Otu0089 Atopobium F20+
Otu0370 Campylobacter F20+
Otu0315 Campylobacter F20+
Otu0142 Campylobacter F20+
Otu0007 Pasteurellaceae_unclassified F8+
Otu0118 Pasteurellaceae_unclassified F20+; F23+
Otu0195 Pasteurellaceae_unclassified F12+
Otu0025 Haemophilus F20+; F23+
Otu0033 Pasteurellaceae_unclassified F14−
Otu0014 Neisseria F20+
Otu0062 Neisseria F20+
Otu0008 Neisseria F20+; F25+
Otu0147 Neisseria F20+
Otu0091 Lautropia F6+
Otu0029 Enterobacteriaceae_unclassified F18−

Effect of OTU clustering on the results

(Supplementary Fig. 11-13, Supplementary Table 2, 5, 7, 8 & table on figshare)

Association between microbiome features and host factors can depend on the phylogenetic resolution at which OTUs are defined[20,37]. To examine the effect of OTU clustering on our conclusions, we compared the results based on ASVs to the results based on the OTUs clustered by *opticlust* or *bdtt* methods, with cutoffs between 0.01 and 0.15.

The effect of urbanization on alpha diversity was overall largely consistent across the clustering methods and cutoffs for both the gut and saliva microbiome (Supplementary Fig. 11). This was also generally the case for the best predictors of the gut microbiome alpha diversity, although the selected set of predictors somewhat varied across the data subsets (whole, rural-urban with and without FFQ data) and diversity indices (Supplementary Table 3, Supplementary Fig. 12). For the saliva microbiome, apart from the overall consistent results obtained for ${}^1D_{ph}$ and ${}^2D_{ph}$, the predictive power of the models based on other indices was low and therefore not considered as reliable for interpretation.

Regarding the gut microbiome community composition, the results were consistent across the clustering methods and cutoffs, with the amount of variation explained by the *ordistep*-selected models slightly higher with increasing cutoff levels (Supplementary Fig. 13). It is noteworthy that the time since the last use of antibiotics was among the most important explanatory variables for some of the higher cutoffs, but not for the fine-scale OTUs and ASVs. Here again, the composition of the saliva microbiome varied in a less predictable manner and the explanatory factors were less consistent across the data subsets, clustering methods and levels (Supplementary Table 5, 7 & 8, Supplementary Fig. 13).

Regarding the relationship between the gut and saliva microbiome, species and lineage richness were weakly but positively correlated for all cutoffs and both methods (mean Pearson $r = 0.23$ and $r = 0.22$, respectively, Supplementary Fig. 9). Although we observed some additional - always weak and positive - correlations for other indices, they varied across the clustering methods and cutoffs. In contrast to ASVs (marginally significant, $p = 0.052$), we found no evidence that the gut and saliva microbiome within individual shared more species than a random gut-saliva microbiome pair when controlling for the urbanization level (but the effect was significant for free permutations, Supplementary Fig. 10).

References

1. Arumugam, M. *et al.* Enterotypes of the human gut microbiome. *Nature* **473**, 174–180 (2011).
2. Holmes, I., Harris, K. & Quince, C. Dirichlet Multinomial Mixtures: Generative Models for Microbial Metagenomics. *PLOS ONE* **7**, e30126 (2012).

3. Ding, T. & Schloss, P. D. Dynamics and associations of microbial community types across the human body. *Nature* **509**, 357–360 (2014).
4. Willis, J. R. *et al.* Citizen science charts two major “stomatotypes” in the oral microbiome of adolescents and reveals links with habits and drinking water composition. *Microbiome* **6**, 218 (2018).
5. Gorvitovskaia, A., Holmes, S. P. & Huse, S. M. Interpreting Prevotella and Bacteroides as biomarkers of diet and lifestyle. *Microbiome* **4**, (2016).
6. Mobeen, F., Sharma, V. & Tulika, P. Enterotype Variations of the Healthy Human Gut Microbiome in Different Geographical Regions. *Bioinformation* **14**, 560–573 (2018).
7. Wu, G. D. *et al.* Linking Long-Term Dietary Patterns with Gut Microbial Enterotypes. *Science* **334**, 105–108 (2011).
8. Vieira-Silva, S. *et al.* Species–function relationships shape ecological properties of the human gut microbiome. *Nature Microbiology* **1**, 16088 (2016).
9. Vandeputte, D. *et al.* Stool consistency is strongly associated with gut microbiota richness and composition, enterotypes and bacterial growth rates. *Gut* **65**, 57–62 (2016).
10. Falony, G. *et al.* Population-level analysis of gut microbiome variation. *Science* **352**, 560–564 (2016).
11. Costea, P. I. *et al.* Enterotypes in the landscape of gut microbial community composition. *Nat Microbiol* **3**, 8–16 (2018).
12. Tyakht, A. V. *et al.* Human gut microbiota community structures in urban and rural populations in Russia. *Nature Communications* **4**, 2469 (2013).
13. Landis, J. R. & Koch, G. G. The Measurement of Observer Agreement for Categorical Data. *Biometrics* **33**, 159–174 (1977).
14. Nasidze, I., Li, J., Quinque, D., Tang, K. & Stoneking, M. Global diversity in the human salivary microbiome. *Genome Res* **19**, 636–643 (2009).
15. Nasidze, I. *et al.* High Diversity of the Saliva Microbiome in Batwa Pygmies. *PLOS ONE* **6**, e23352 (2011).

16. Li, J. *et al.* Comparative analysis of the human saliva microbiome from different climate zones: Alaska, Germany, and Africa. *BMC Microbiology* **14**, 316 (2014).
17. Lassalle, F. *et al.* Oral microbiomes from hunter-gatherers and traditional farmers reveal shifts in commensal balance and pathogen load linked to diet. *Molecular Ecology* (2017) doi:10.1111/mec.14435.
18. Tikhonov, M., Leach, R. W. & Wingreen, N. S. Interpreting 16S metagenomic data without clustering to achieve sub-OTU resolution. *The ISME Journal* **9**, 68–80 (2015).
19. Callahan, B. J., McMurdie, P. J. & Holmes, S. P. Exact sequence variants should replace operational taxonomic units in marker-gene data analysis. *The ISME journal* **11**, 2639 (2017).
20. Graham, C. H., Storch, D. & Machac, A. Phylogenetic scale in ecology and evolution. *Global Ecology and Biogeography* **27**, 175–187 (2018).
21. Washburne, A. D. *et al.* Phylofactorization: a graph partitioning algorithm to identify phylogenetic scales of ecological data. *Ecological Monographs* e01353 (2019) doi:10.1002/ecm.1353.
22. Pinheiro, J., Bates, D., DebRoy, S., Sarkar, D. & R Core Team. *nlme: Linear and Nonlinear Mixed Effects Models*. (2019).
23. Mancabelli, L. *et al.* Meta-analysis of the human gut microbiome from urbanized and pre-agricultural populations: The urbanization/industrialization of humans and gut microbiomes. *Environmental Microbiology* **19**, 1379–1390 (2017).
24. Precup, G. & Vodnar, D.-C. Gut Prevotella as a possible biomarker of diet and its eubiotic versus dysbiotic roles: a comprehensive literature review. *British Journal of Nutrition* 1–10 (undefined/ed) doi:10.1017/S0007114519000680.
25. Zhernakova, A. *et al.* Population-based metagenomics analysis reveals markers for gut microbiome composition and diversity. *Science* **352**, 565–569 (2016).
26. Das, B. *et al.* Analysis of the Gut Microbiome of Rural and Urban Healthy Indians Living in Sea Level and High Altitude Areas. *Scientific Reports* **8**, 10104 (2018).

27. De Filippis, F., Pellegrini, N., Laghi, L., Gobbetti, M. & Ercolini, D. Unusual sub-genus associations of faecal Prevotella and Bacteroides with specific dietary patterns. *Microbiome* **4**, 57 (2016).
28. Cohan, F. & Koeppel, A. The origins of ecological diversity in prokaryotes. *Curr Biol* **18**, R1024-34 (2008).
29. Menni, C. *et al.* Gut microbiome diversity and high-fibre intake are related to lower long-term weight gain. *International Journal of Obesity* **41**, 1099–1105 (2017).
30. Okeke, F., Roland, B. C. & Mullin, G. E. The Role of the Gut Microbiome in the Pathogenesis and Treatment of Obesity. *Glob Adv Health Med* **3**, 44–57 (2014).
31. Zaiss, M. M. *et al.* The Intestinal Microbiota Contributes to the Ability of Helminths to Modulate Allergic Inflammation. *Immunity* **43**, 998–1010 (2015).
32. Holm, J. B. *et al.* Chronic Trichuris muris Infection Decreases Diversity of the Intestinal Microbiota and Concomitantly Increases the Abundance of Lactobacilli. *PLOS ONE* **10**, e0125495 (2015).
33. Shin, J.-H., Sim, M., Lee, J.-Y. & Shin, D.-M. Lifestyle and geographic insights into the distinct gut microbiota in elderly women from two different geographic locations. *Journal of Physiological Anthropology* **35**, 31 (2016).
34. Brahe, L. K. *et al.* Specific gut microbiota features and metabolic markers in postmenopausal women with obesity. *Nutr & Diabetes* **5**, e159–e159 (2015).
35. Kaakoush, N. O. Insights into the Role of Erysipelotrichaceae in the Human Host. *Front Cell Infect Microbiol* **5**, (2015).
36. Lu, M., Xuan, S. & Wang, Z. Oral microbiota: a new view of body health. *Food Science and Human Wellness* (2019) doi:10.1016/j.fshw.2018.12.001.
37. Groussin, M. *et al.* Unraveling the processes shaping mammalian gut microbiomes over evolutionary time. *Nature Communications* **8**, 14319 (2017).

Supplementary Figures

Supplementary Fig. 1. Strong correlations between the collected contextual variables and multivariate analysis (FAMD) of non-dietary data,

Supplementary Fig. 2. Multivariate analysis (PCA) of 24h-recall dietary data.

Supplementary Fig. 3. Multivariate analysis (PCA) of FFQ dietary data.

Supplementary Fig. 4. The most important predictors of ASV diversity for the rural-urban data subset selected by random forest regression.

Supplementary Fig. 5. Major phylofactors in the gut and saliva microbiome associated with the urbanization gradient.

Supplementary Fig. 6. Multivariate analysis (PCA) of the gut and saliva microbiome composition and associated explanatory variables.

Supplementary Fig. 7. Enterotypes and stomatotypes identified by Dirichlet multinomial modelling.

Supplementary Fig. 8. Best predictors of enterotypes and stomatotypes identified by random forest classification.

Supplementary Fig. 9. Correlation between diversity of the gut and saliva microbiome of the same individual.

Supplementary Fig. 10. Are the gut and saliva microbiome of the same individual more similar to each other than a random gut-saliva microbiome pair?

Supplementary Fig. 11. Effect of OTU clustering on the relationship between alpha diversity and urbanization.

Supplementary Fig. 12. Effect of OTU clustering on the selection of alpha diversity predictors.

Supplementary Fig. 13. Effect of OTU clustering on the amount of variance explained and the variables explaining the variation in the gut and saliva microbiome composition.

Supplementary Fig. 1. Strong correlations between the collected contextual variables and multivariate analysis (FAMD) of non-dietary data. Upper row: strong correlations (pearson >=0.8 or omega and Cramer's V>=0.4) between the contextual variables for the whole dataset (left) and for the rural-urban subset with FFQ (food frequency questionnaire) data (right); dr = 24h-recall dietary variables. **Middle row:** FAMD showing individuals (left) and non-dietary variables (right) significantly contributing to the construction of the first and the third FAMD axes; ordered factors were coded both as numerical (num.) and categorical (cat.) variable (see also Fig. 1). **Lower row:** non-dietary variables ordered by their contribution to the construction of FAMD axes. Red line represents the expected value if the distribution was uniform and the variables above this line are considered as significantly contributing to the axis construction.

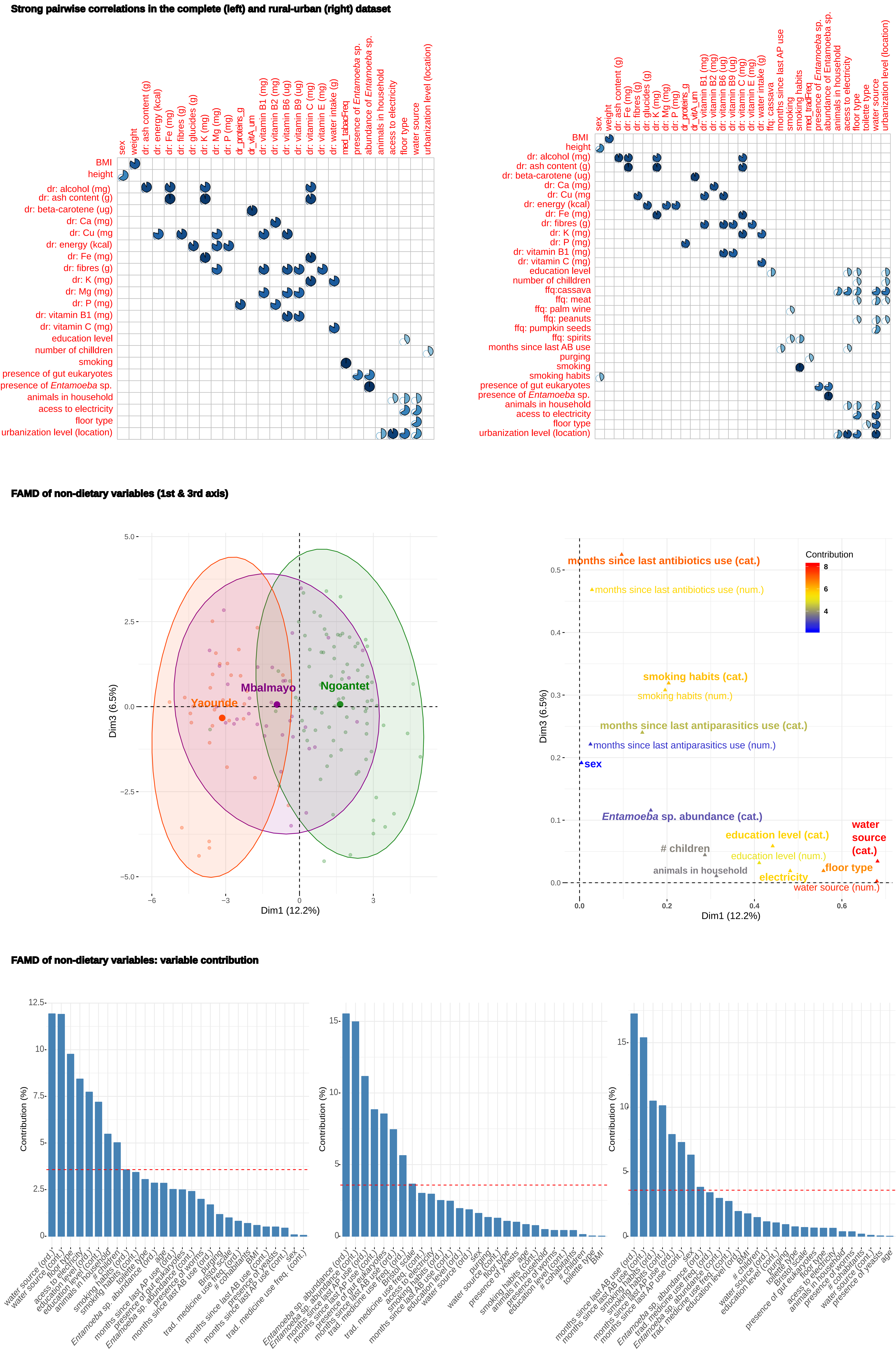

Supplementary Fig. 2. Multivariate analysis (PCA) of 24h-recall dietary data. PCA showing the individuals colored by urbanization level (A, C) and the variables that contributed to the construction of the axes more than expected by chance (B, D) for the first and the second (upper row) and the first and the third (lower row) axis combinations.

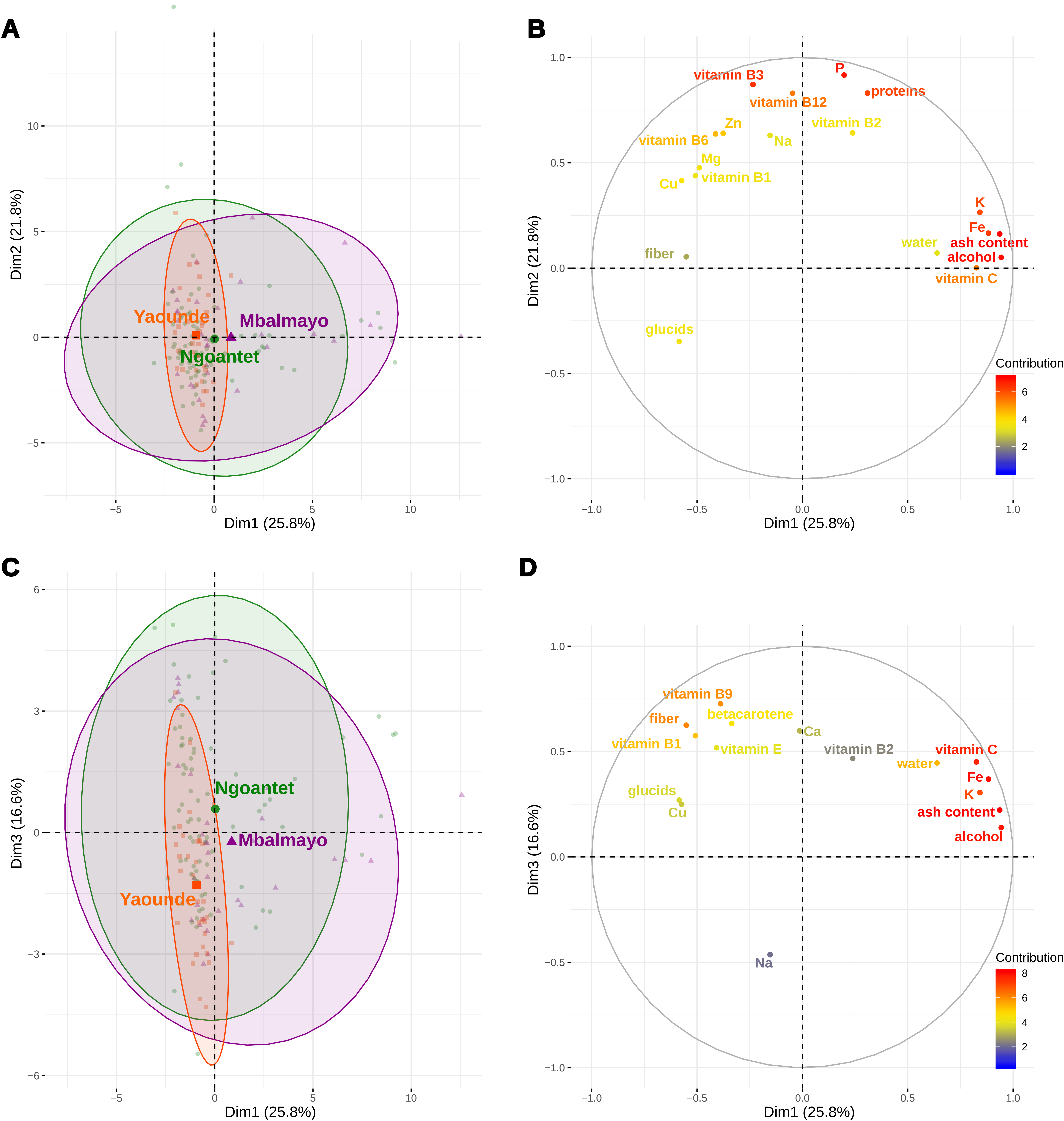

Supplementary Fig. 3. Multivariate analysis (PCA) of FFQ dietary data. PCA showing the individuals colored by urbanization level (A, C) and the FFQ variables that contributed to the construction of the axes more than expected by chance (B, D), for the first and the second (upper row) and the first and the third (lower row) axis combinations. E) Dietary items signficantly enriched in rural (green) or urban (red) environment, tested by v test.

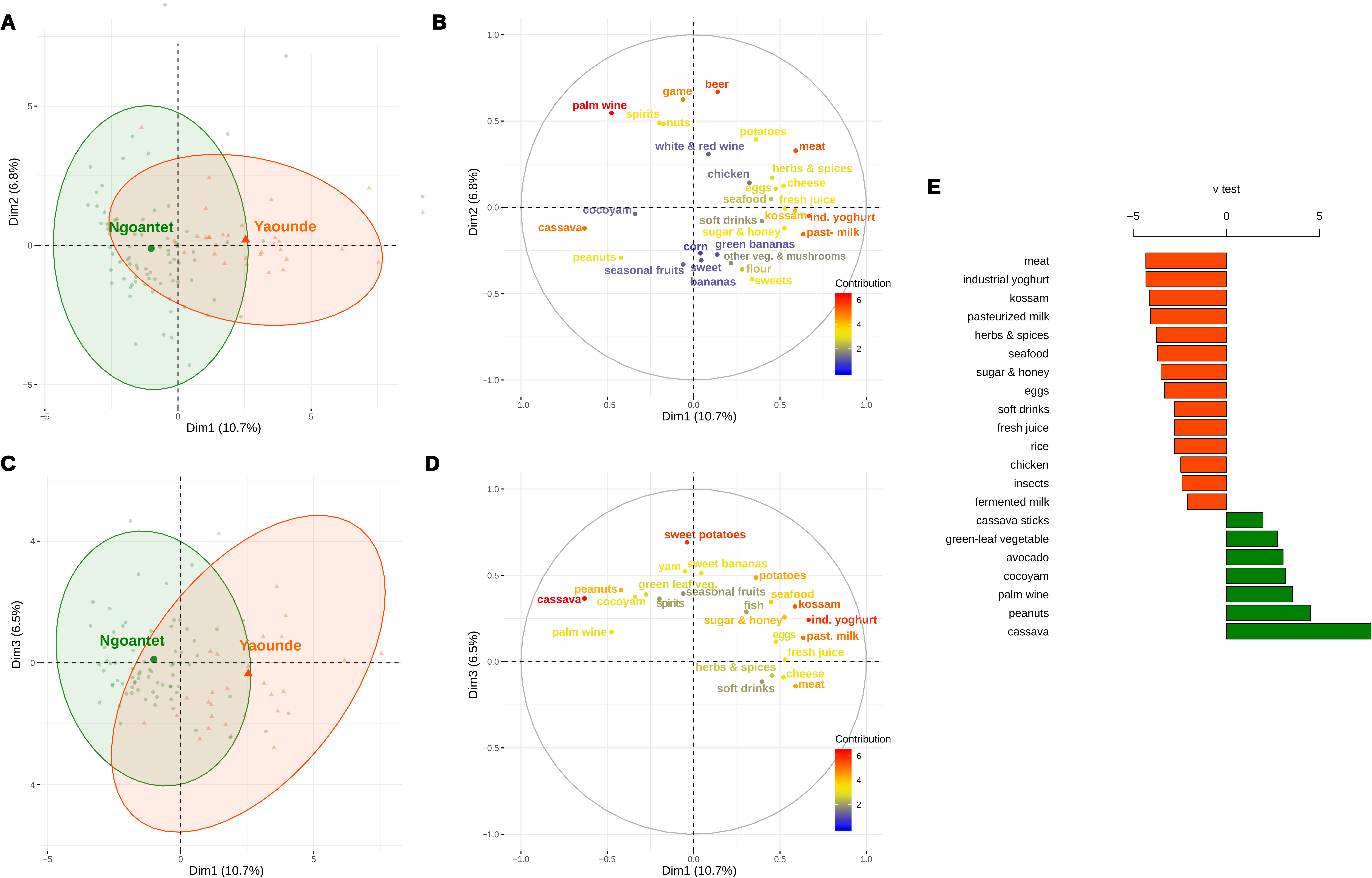

Supplementary Fig. 4. The most important predictors of ASV diversity for the rural-urban data subset selected by random forest regression. Results for the gut and saliva microbiome with (**A, C**) and without (**B, D**) FFQ data included. The values represent the mean of 10 model replicates. Depicted variables were selected by kmeans clustering of the variable importance values as described in Methods section, only for the models with R-squared > 0.1. Index explanations can be found in Table 1. See also Fig. 3. and Supplementary Table 3**.**

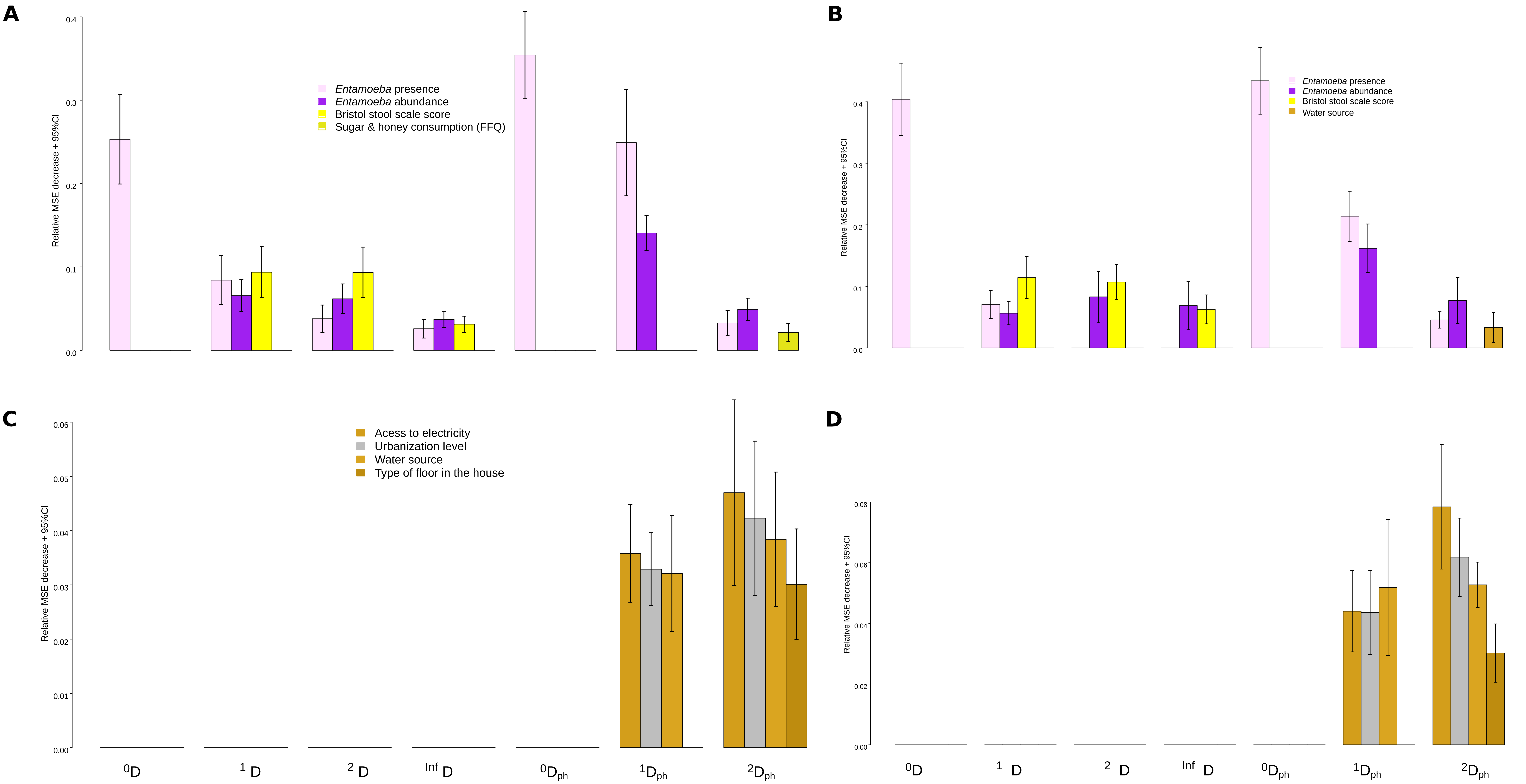

Supplementary Fig. 5. Major phylofactors in the gut and saliva microbiome associated with the urbanization gradient. Phylofactors are taxa or group of taxa that best differentiate between the tested groups, in this case between the urbanization levels (see also Supplementary Results). Pairs of plots for the gut microbiome phylofactors are on the left, for the saliva microbiome phylofactors on the right. The left plot in the pair shows the predicted change in relative abundance of the phylofactor on the log scale, the plot on the right shows the observed ratio of geometric means of the phylofactor vs. the rest of the community on the original scale.The taxon name is the lowest common ancestor of the taxa in the given phylofactor. Only the first six phylofactors are shown for each microbiome type

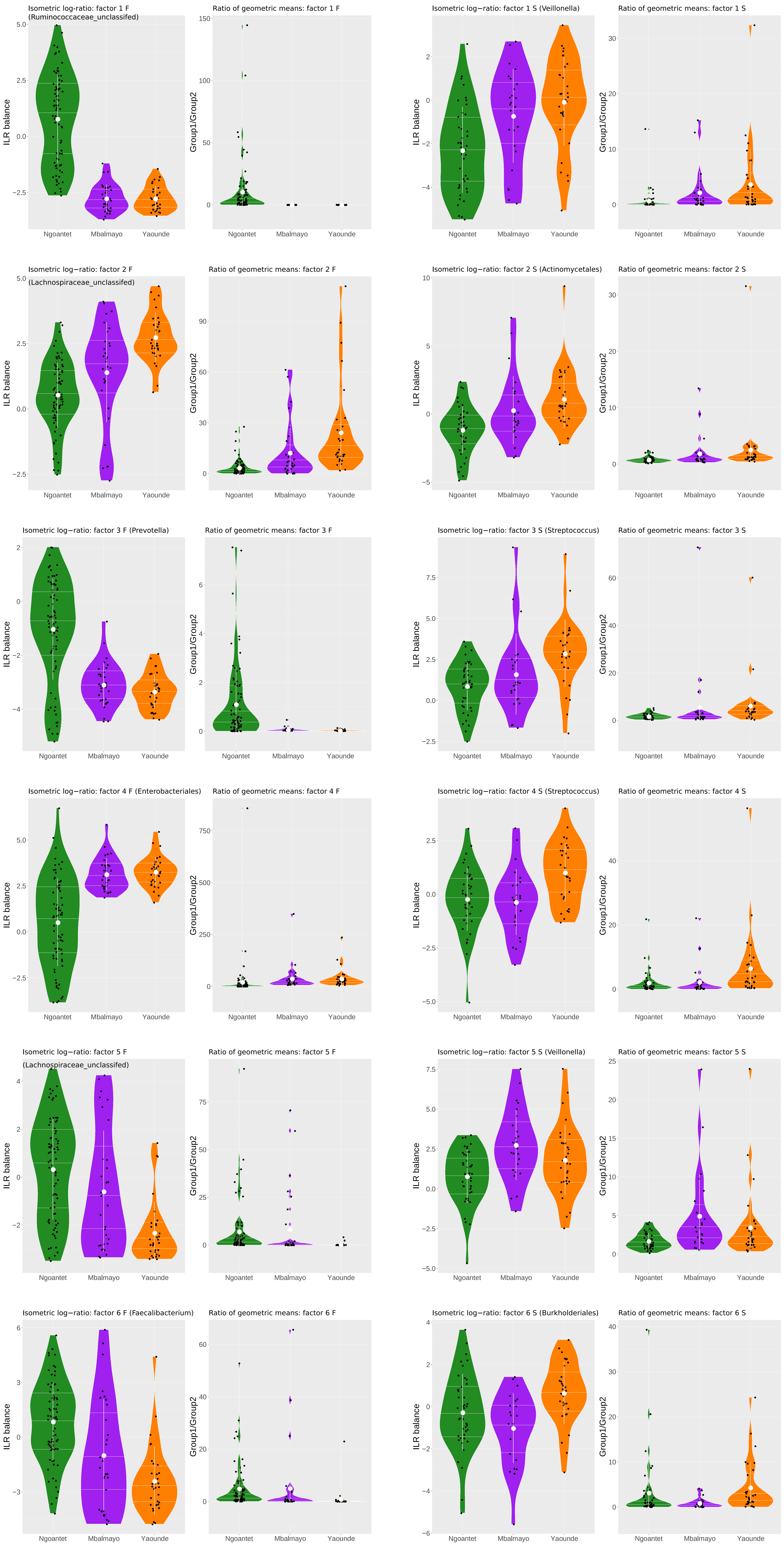

Supplementary Fig. 6. Multivariate analysis (PCA) of the gut and saliva microbiome composition and associated explanatory variables. PCA of Aitichison equivalent distances for the gut (**A, B, E-H**) and saliva microbiome (**C, D, I-L**). **A-D** show the first and the third axis of the microbiome variation for the whole dataset (see also Fig. 4 and Fig. 5), **E-L** show the first three axes of varitarion in he rural-urban data subset only. Individuals colored by urbanization level and shapes representing the community types (enterotypes or stomatotypes, see Supplementary Results) are depicted in lthe left column, contextual variables selected by *ordistep* and ASVs associated either with these variables or with the ordination axes (only the ASVs and variables within the lower or upper 2.5% quantile are shown, for all variables (see Supplementary Table 7-8) are shown in the right column. ASVs and numerical variables are represented by arrows, factors are represented by their levels' names placed at the centroid of individuals of the given category. **M-P** Variation in community composition explained by different factors selected by *envfit* (right, each variable tested independently) and *ordistep* (left, the factors retained in the best non-redundant model) for the gut (**M-N**) and saliva (**O-P**) microbiome in the rural-urban data subset; dr = 24h-recall, ffq = food frequency questionnaire. Compete results for each ASV, explanatory variable and clusting method can be found in Supplementary Table 7-8.

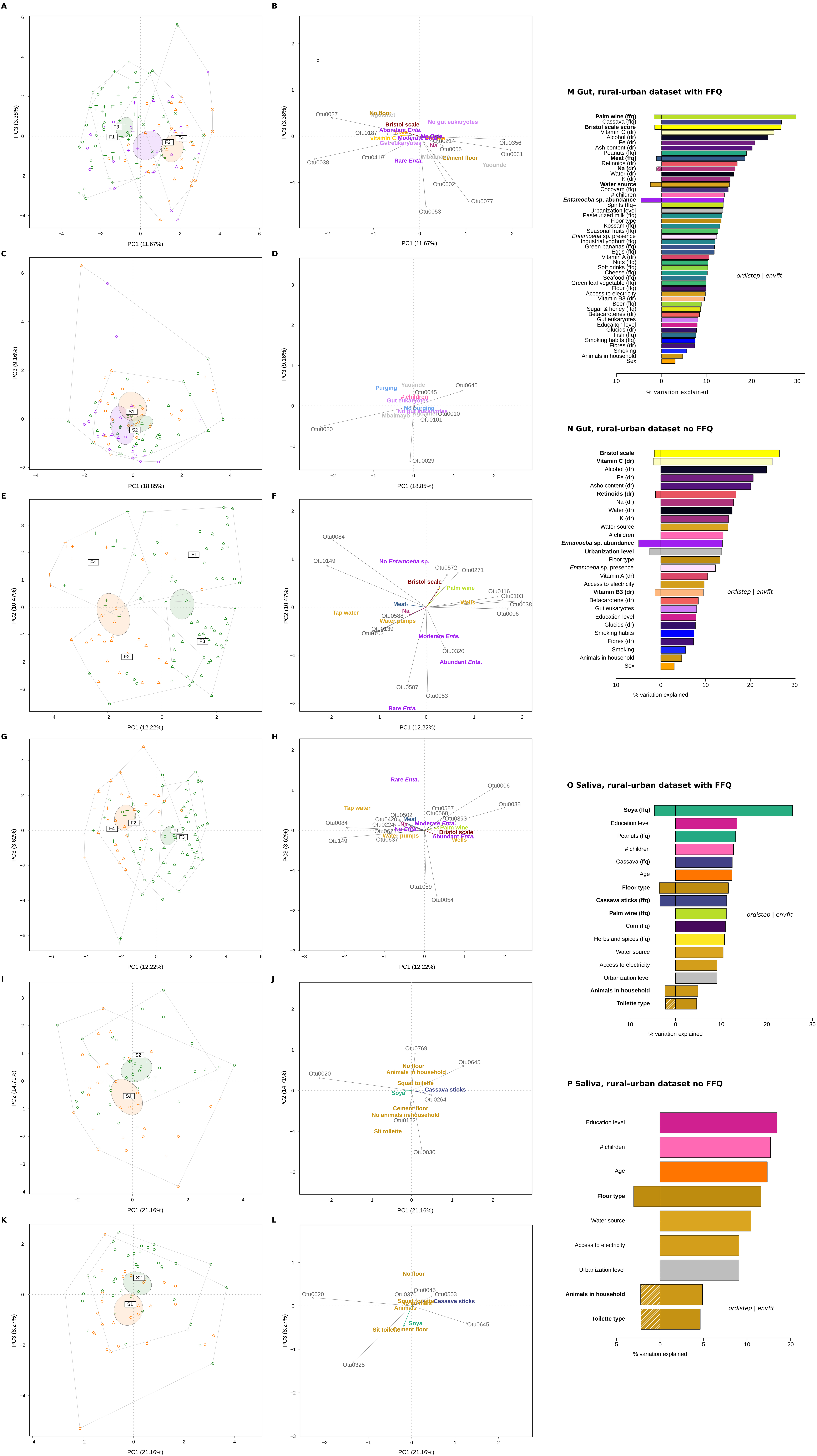

Supplementary Fig. 7. Enterotypes and stomatotypes identified by Dirichlet multinomial modelling. A) Enterotypes (gut) and B) stomatotypes (saliva) identified by Dirichlet multinomial models of genus-level agglomerated data, showing the taxa accounting for 90% of the difference between the chosen and the null model. The broader columns represent the predicted community type and the narrower columns represent the individual samples. Additional information on community types can be found in Supplementary Fig. 8 and Supplementary Results.

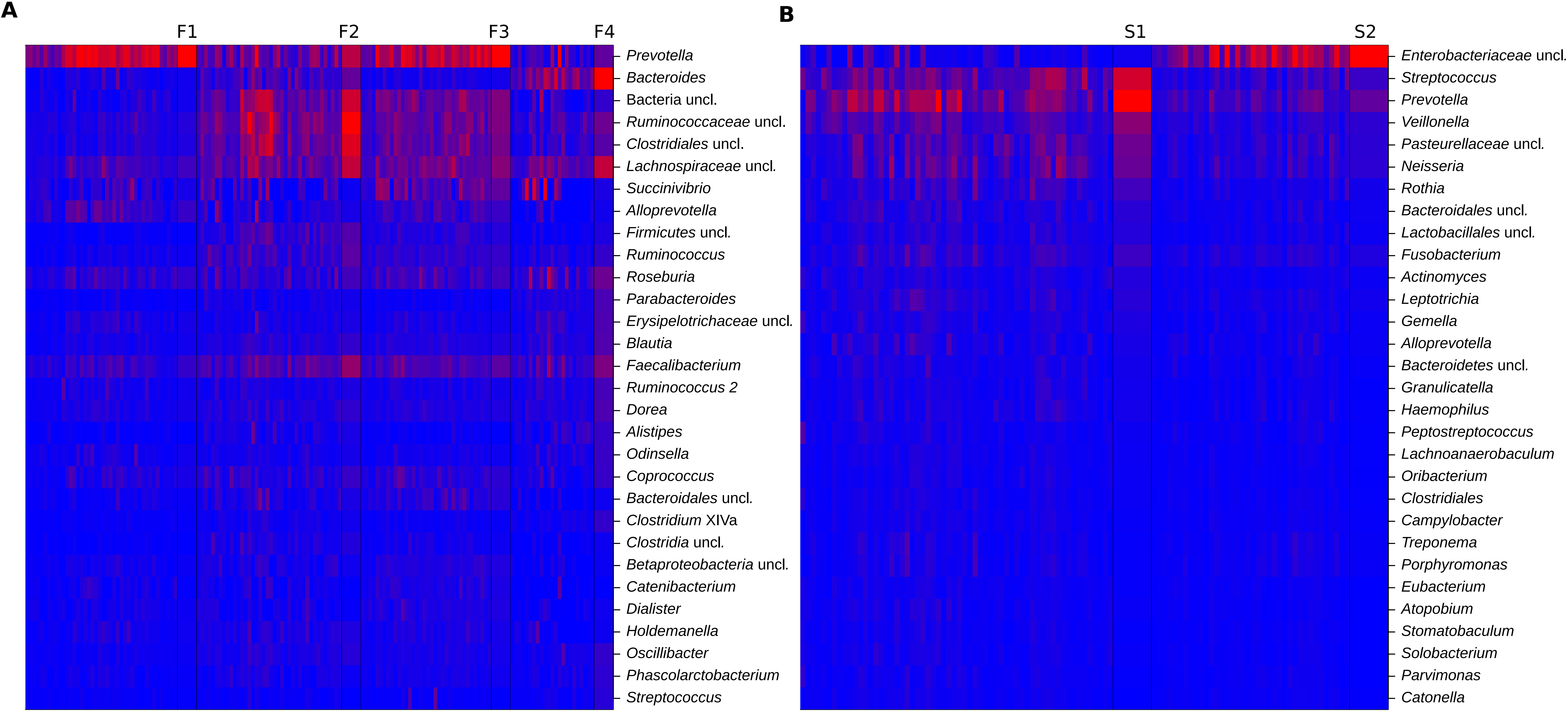

Supplementary Fig. 8. Best predictors of enterotypes and stomatotypes identified by random forest classification. **A**) Kappa and **B**) accuracy values for community types of both microbiomes, for all three data subsets. The values were acalculated either on a separated test data (pink) or as out-of-bag estimates (green). Only the models with test Kappa >=0.3 were considered for interpretation. Variable importance for enterotype (**C, E, G**) and stomatotype (**D, F, H**) prediction in the complete (**C, D**) and rural-urban datasets with (**E, F**) and without FFQ (**G, H**) data, respectively. Only the variables with mean importance higher than a random variable (see Methods) are shown. The significant variables, i.e. the variables selected by kmeans clustering are shown in red bold. Community types area represented in Supplemenatry Fig. 7 and described in detail in Supplementary Results.

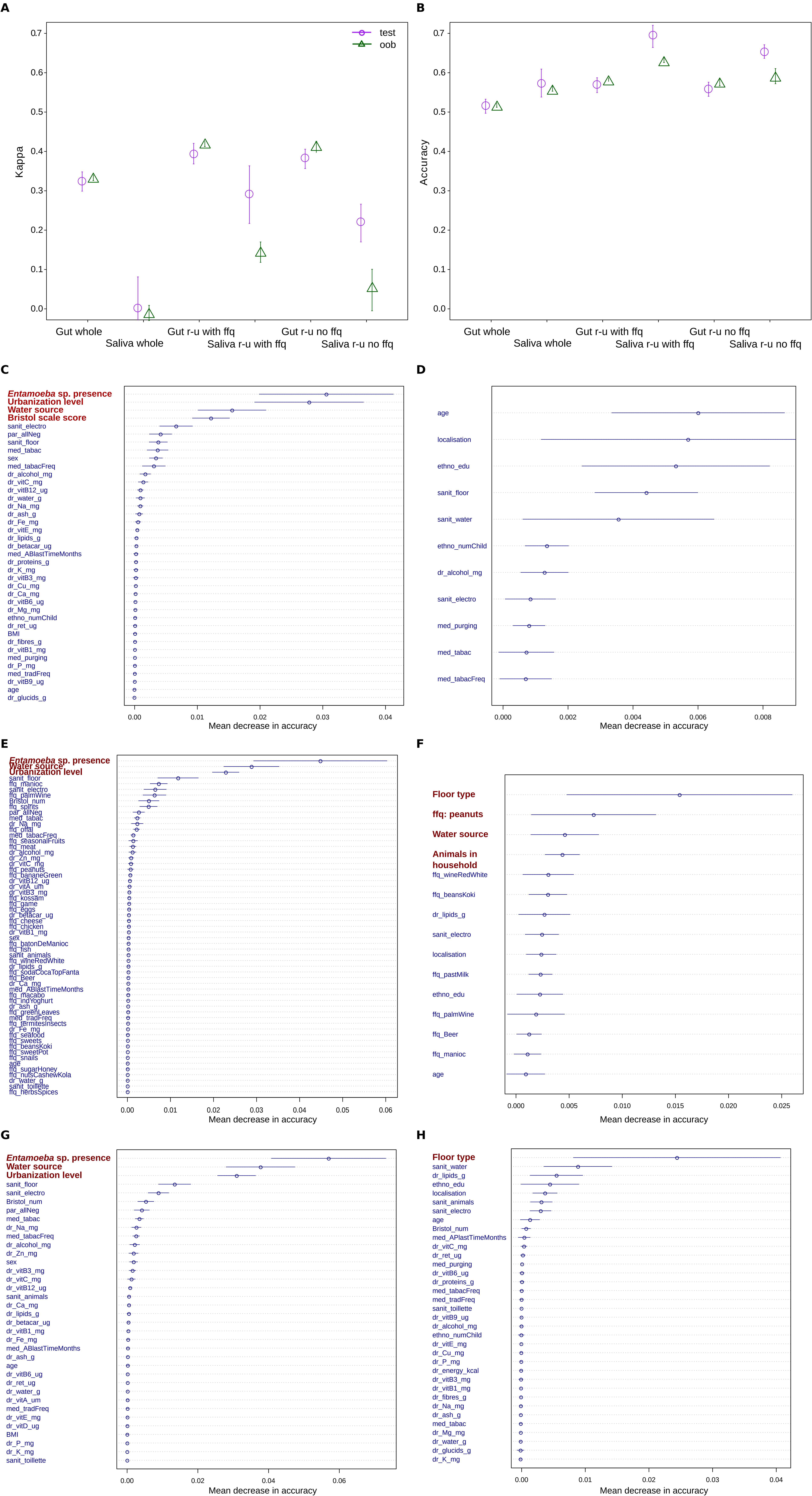

Supplementary Fig. 9. Correlation between diversity of the gut and saliva microbiome of the same individual. Pearson correlations for all alpha diversity indices listed in Table 1, for both clustering methods and all cutoffs.

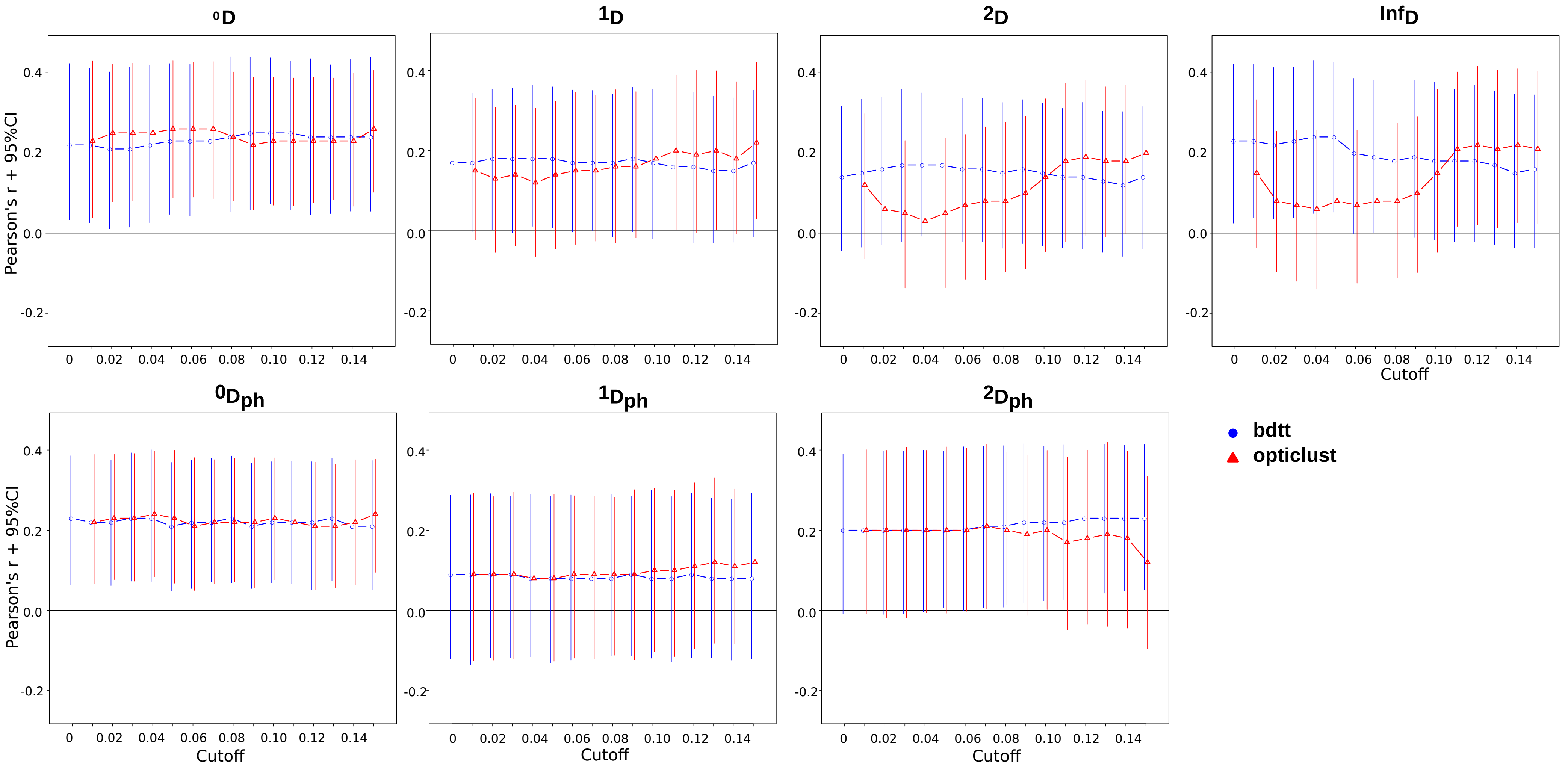

Supplementary Fig. 10. Are the gut and saliva microbiome of the same individual more similar to each other than a random gut-saliva microbiome pair? A) p-values and standardized effect size expressing if and how much the gut and saliva microbiome of a single individual are more similar (in terms of taxon turnover) to each other than a random gut-saliva pair (based on 1000 free permutations); **B)** p-values and standardized effect size expressing if and how much the gut and saliva microbiome of a single individual are more similar to each other than a random gut-saliva pair in the same(rural, semi-urban, urban) population; **C)** relationship between log10 (p-value) for the free and urbanization level constrained results; **D)** relationship between the standardized effect sizes for the free and urbanization-level constrained results. Significant effect sizes are in red. The diagonal line in C-D represents perfect correlation.

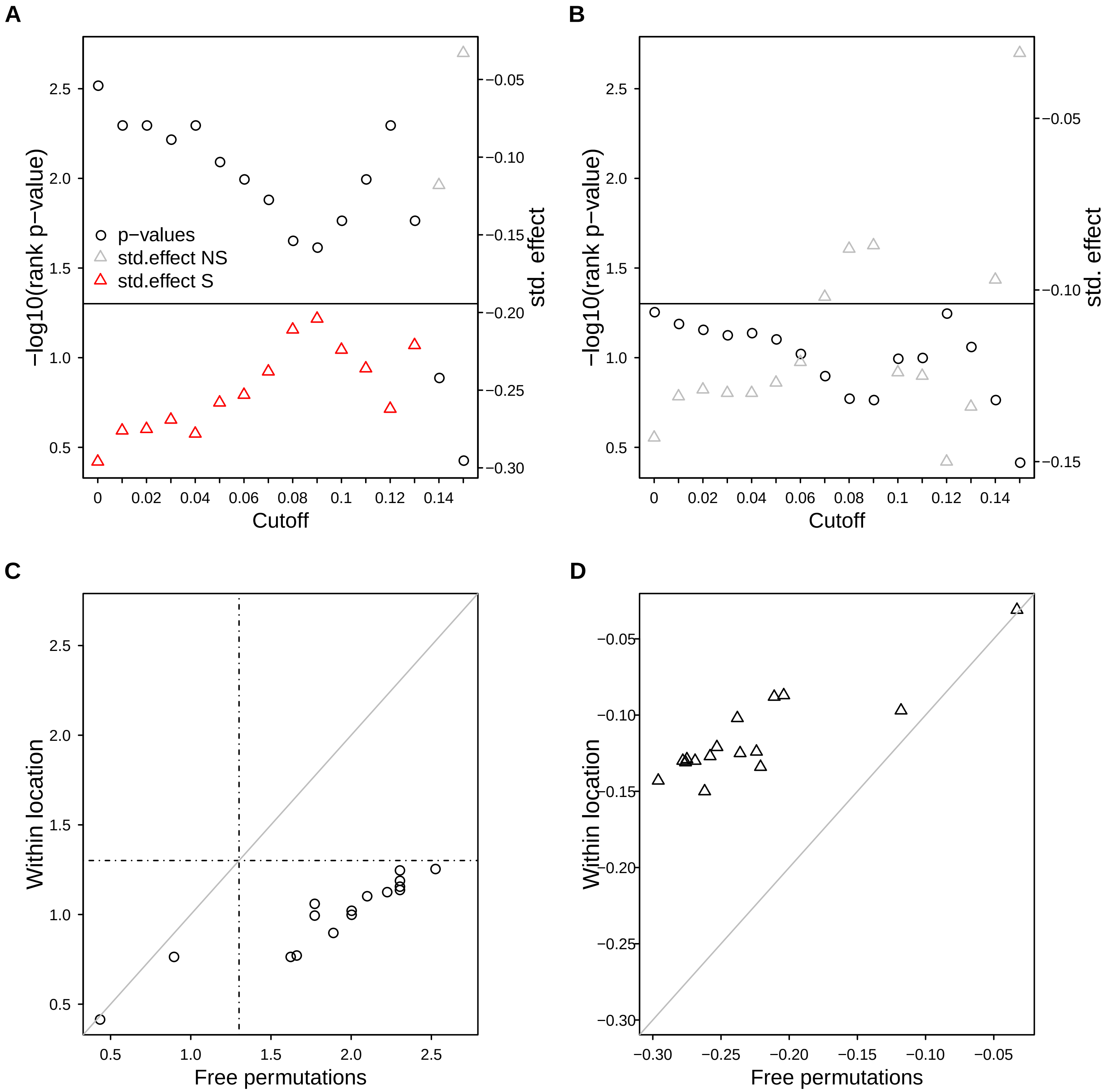

Supplementary Fig. 11. Effect of OTU clustering on the relationship between alpha diversity and urbanization. Urbanization gradient is represented by linear polynomial contrast ("linear trend"). Bold points denote significant effects. Points represent % change with 95 % confidence intervals.

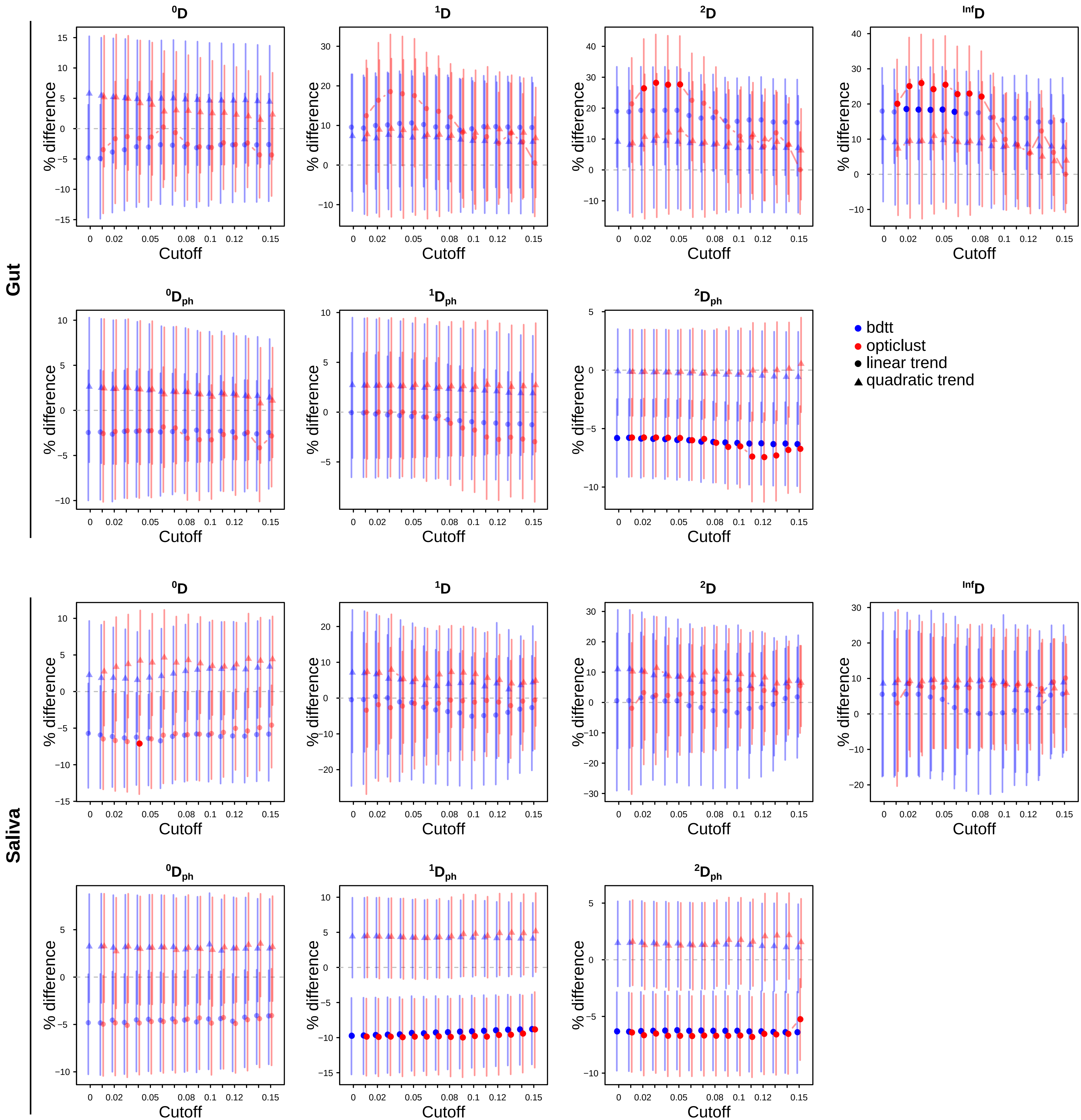

Supplementary Fig. 12. Effect of OTU clustering on the selection of alpha diversity predictors. Best predictors of alpha diversity by random forest regression across the clustering methods and cutoffs. The plots show the overlap (or lack of it) between the significant predictors (selected by kmeans clustering of random forest importances, see Methods) of alpha diversity across the clustering methods and cutoffs. Only the predictors from models with R2 > 0.1 were considered for interpretation. **Columns** represent the results for the 1) whole dataset and for the rural-urban subset 2) with and 3) without FFQ (food frequency questionnaire data included). First six rows show the results for the gut, the last six for the saliva microbiome (each row shows the results for one index). If there is a single multicolored column - the results are completely consistent for all the significant models (R2>=0.1) across the methods and cutoffs. Note that very few models are significant for non-phylogenetic indices for the saliva microbiome. See also Supplementary Table 3 and the table on figshare linked with this study.

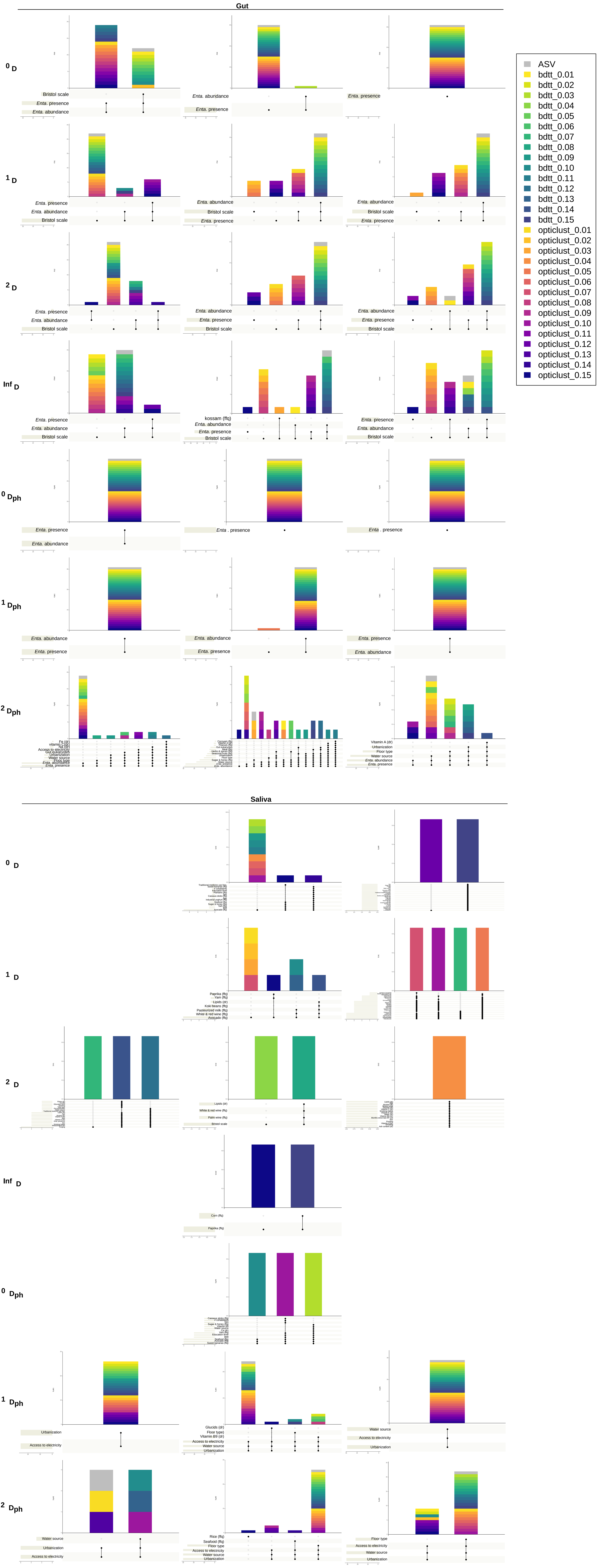

Supplementary Fig. 13. Effect of OTU clustering on the amount of variance explained and the variables explaining the variation in the gut and saliva microbiome composition. Variance explained by the *ordistep*-selected best model across clustering methods and cutoffs for the gut (left) and saliva (right) microbiome. Two plots are available for each data subset (whole, rural-urban with and without FFQ): the one in the first row shows the effect of individual variables, the one in the second row shows variables grouped by type (e.g. medical, dietary…). Each column represents one of the datasets obtained by two clustering methods (opticlust and bdtt) for clustering cutoffs 0.01-0.15 at 0.01 clustering steps as described in Methods. The first column represents the ASV dataset on which the main conclusions of the study are based. The variables are ordered by their contribution to the explained variation for each method and cutoff separately. dr = 24h-recall data.

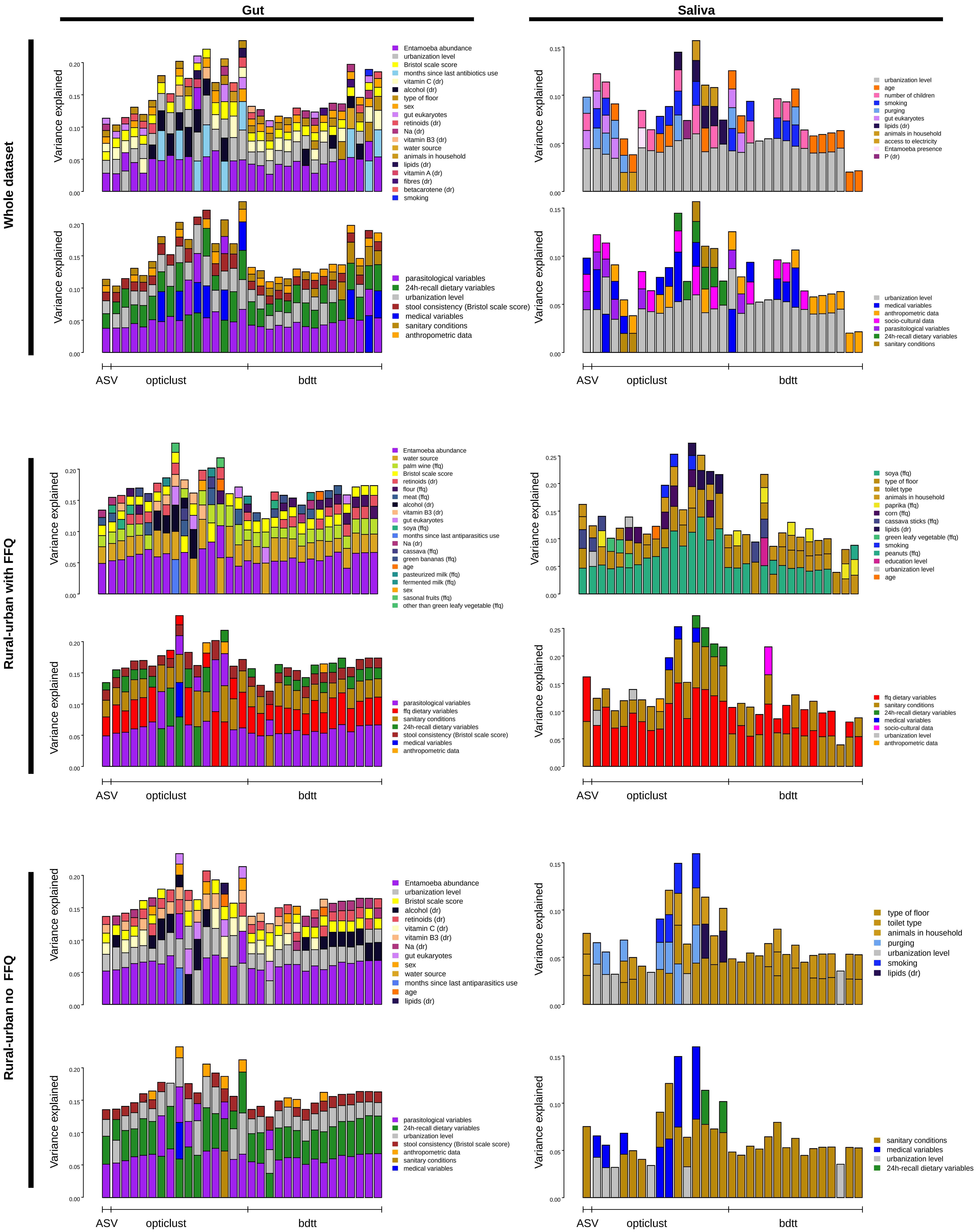

List of supplementary tables

The tables are available in a separate *xls file.

Supplementary Table 1. Contextual information about the sampled individuals.

Supplementary Table 2. Alpha diversity of the gut and saliva microbiome along the urbanization gradient.

Supplementary Table 3. Best predictors of alpha diversity identified by random forest regression.

Supplementary Table 4. Correlations between the collected contextual variables (metadata); Supplementary Table 5. Differences in ASV/OTU abundances along the urbanization gradient (ALDEx2).

Supplementary Table 6. Phylofactors associated with the urbanization gradient for the gut and saliva microbiome.

Supplementary Table 7. ASVs and OTUs associated with the variation in the gut and saliva microbiome composition or metadata.

Supplementary Table 8. PCA coordinates of the variables selected by *envfit* for each data subset and microbiome type. **Supplementary Table 9.** Effect of quality control on number of reads and unique sequences in total and per sample.

Supplementary Table 10. Number of OTUs and total number of reads across the clustering methods and cutoffs.
